# *Babela massiliensis*, a representative of a widespread bacterial phylum with unusual adaptations to parasitism in amoebae

**DOI:** 10.1186/s13062-015-0043-z

**Published:** 2015-03-31

**Authors:** Isabelle Pagnier, Natalya Yutin, Olivier Croce, Kira S Makarova, Yuri I Wolf, Samia Benamar, Didier Raoult, Eugene V Koonin, Bernard La Scola

**Affiliations:** URMITE, CNRS UMR IRD 6236, Faculté de Médecine, Université de la Méditerranée, 27 Bd. Jean Moulin, 13385 Marseille Cedex 5, France; National Center for Biotechnology Information, National Library of Medicine, National Institutes of Health, Bethesda, MD 20894 USA

**Keywords:** Intracellular bacteria, Amoeba, Bacterial replication

## Abstract

**Background:**

Only a small fraction of bacteria and archaea that are identifiable by metagenomics can be grown on standard media. Recent efforts on deep metagenomics sequencing, single-cell genomics and the use of specialized culture conditions (culturomics) increasingly yield novel microbes some of which represent previously uncharacterized phyla and possess unusual biological traits.

**Results:**

We report isolation and genome analysis of *Babela massiliensis*, an obligate intracellular parasite of *Acanthamoeba castellanii. B. massiliensis* shows an unusual, fission mode of cell multiplication whereby large, polymorphic bodies accumulate in the cytoplasm of infected amoeba and then split into mature bacterial cells. This unique mechanism of cell division is associated with a deep degradation of the cell division machinery and delayed expression of the *ftsZ* gene. The genome of *B. massiliensis* consists of a circular chromosome approximately 1.12 megabase in size that encodes, 981 predicted proteins, 38 tRNAs and one typical rRNA operon. Phylogenetic analysis shows that *B. massiliensis* belongs to the putative bacterial phylum TM6 that so far was represented by the draft genome of the JCVI TM6SC1 bacterium obtained by single cell genomics and numerous environmental sequences.

**Conclusions:**

Currently, *B. massiliensis* is the only cultivated member of the putative TM6 phylum. Phylogenomic analysis shows diverse taxonomic affinities for *B. massiliensis* genes, suggestive of multiple gene acquisitions via horizontal transfer from other bacteria and eukaryotes. Horizontal gene transfer is likely to be facilitated by the cohabitation of diverse parasites and symbionts inside amoeba. *B. massiliensis* encompasses many genes encoding proteins implicated in parasite-host interaction including the greatest number of ankyrin repeats among sequenced bacteria and diverse proteins related to the ubiquitin system. Characterization of *B. massiliensis*, a representative of a distinct bacterial phylum, thanks to its ability to grow in amoeba, reaffirms the critical role of diverse culture approaches in microbiology.

**Reviewers:**

This article was reviewed by Dr. Igor Zhulin, Dr. Jeremy Selengut, and Pr Martijn Huynen.

**Electronic supplementary material:**

The online version of this article (doi:10.1186/s13062-015-0043-z) contains supplementary material, which is available to authorized users.

## Background

Since the onset of the genome sequencing era, evolution of obligate intracellular bacteria has been viewed in a sharp contrast to the evolution of free-living bacteria, especially those that inhabit complex environments. Intracellular bacteria, such as *Chlamydiae* or *Rickettsiae*, were generally regarded as organisms characterized by extreme adaptation to a narrow ecological niche (the host cell) associated with a massive gene loss resulting in dramatic genome reduction [1-3]. Horizontal gene transfer (HGT) was believed to be infrequent in these bacteria, despite some apparent important exceptions that could be linked to specific adaptations for the intracellular lifestyle such as the apparent transfer of the ATP/ADP translocase gene from eukaryotes to *Chlamydiae* and *Rickettsiae* [4,5]. In contrast, free-living bacteria, especially those that inhabit environments with diverse microbiota, such as soil or the gut of vertebrates, are subject to extensive HGT [6] which leads to highly variable genomic content even among bacteria that are considered to be closely related. For example, in a comparative analysis of 61 sequenced genomes of *E. coli*, the core genome common to all analyzed strains comprised only about 6% of the gene families [7].

Recently, however, the notion of the fundamental distinction between the evolutionary modalities of intracellular and extracellular bacteria was replaced by the more biologically realistic concept of allopatric vs. sympatric lifestyles [7-9]. Specifically, in amoebae, intracellular bacteria are not genetically isolated but rather coexist sympatrically with other bacteria, archaea and NCLDV (Nucleocytoplasmic large DNA viruses), specifically amoeba-associated giant viruses, a lifestyle that is conducive to HGT [10-12]. Thanks to their capacity to engulf any large particle and thus to bring into proximity numerous, diverse microbes, amoebae appear to be a melting pot of evolution from which new genes, new associations of genes and new life forms have been emerging throughout the course of evolution [12].

In the course of isolation of giant viruses, we have serendipitously discovered an apparently several strictly intra-amoebal bacteria. This report describes a bacterium that we denoted *Babela massiliensis*. This bacterium shows a mode of multiplication that, to our knowledge, has not been so far identified in prokaryotes. We show that *B. massiliensis* is the only cultured bacterium from the putative TM6 phylum, for which only one, 90% complete genome, JCVI TM6SC1, has been reported through single cell genomics [13], but that apparently is extremely widespread in diverse habitats. We also describe a variety of genes that appear to represent specific adaptations of *B. massiliensis* to the intra-amoebal lifestyle.

## Results

### The unique morphology of *Babela massiliensis*

**Figure 1:**
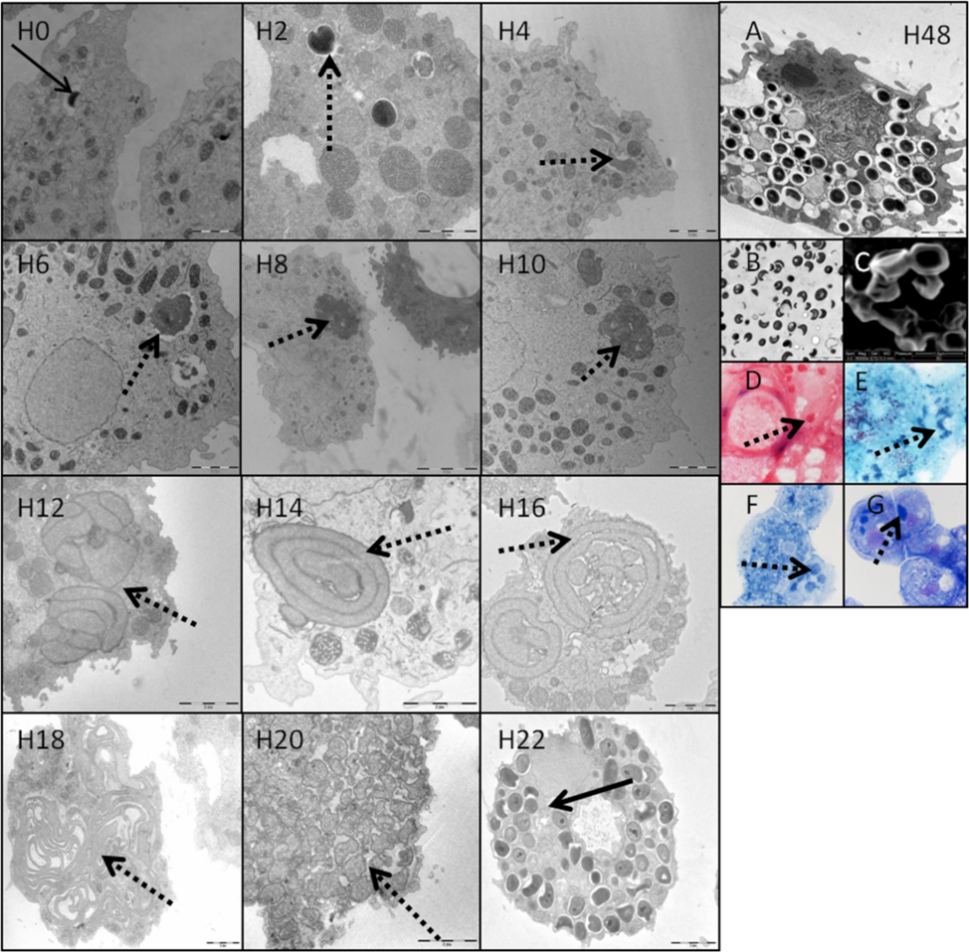
**Morphology and replicative cycle of** ***Babela massiliensis.*** Left side: replicative cycle of *B. massiliensis* in *Acanthamoeba castellanii*, observed from H0 to H22 pi, with transmission electron microscopy. Solid arrows indicate the mature bacterial particles, and dotted arrows indicate the amorphous immature bacterial forms. Right side: observation of the mature forms of the bacteria at H48pi (**A** = transmission electron microscopy of an amoeba infected with mature bacterial particles, **B** = transmission electron microscopy of culture supernatant containing mature particles outside the amoeba, **C** = scanning electron microscopy, **D** = Gram staining, **E** = Gimenez staining, **F** = methyl blue staining, **G** = hemacolor staining).

In amoeba co-culture after 48 hours, we performed a Gram staining, in order to determinate if the bacteria belong to Gram negative or Gram positive group; and Gimenez staining, to assess the intracellular nature of the bacteria [14]. We observed the cytoplasm of the amoeba filled with small Gram negative, Gimenez intracellular positive cocci of a bacterium that we named *Babela massiliensis*. Methyl blue and Hemacolor (Merck-Millipore) staining were also performed in order to see the intracellular organization of both bacteria and amoebal intracytoplasmic features, such as the nucleus and vacuoles. After 48 hours post-infection, the bacteria-infested amoeba also contained amorphous stained structures, with the appearance of dark pink, dark green, dark blue and dark purple spots that were detected within the cytoplasm of the amoeba by using, respectively, Gram, Gimenez, Methyl blue and Hemacolor staining (Figure 1D-E-F-G). Electron microscopy of both infected amoeba and culture supernatant revealed mature forms of the bacteria with an identical morphology, i.e. crescent and round bodies (Figure 1A and B). Scanning electron microscopy suggests that the bacteria have the shape of a bowl (Figure 1C) which explains the two forms observed in electron micrographs of thin sections. The bacteria seem to be strictly intracellular and apparently unable to grow outside amoeba; of all axenic conditions tested, such as several agar media and nutritive broths (PYG, Trypticase Soy broth), under aerobic, anaerobic and microaerophilic atmospheres, none allowed bacterial growth. Among the temperatures tested for growth in amoebal coculture, *B. massiliensis* was unable to grow at 4°C and 12°C but showed growth at 28°C and 37°C. In this respect, *B. massiliensis* resembles *Parachlamydia acanthamoeba*, another strict intra-amoebal bacterium [15].

### Unique developmental cycle

In the beginning of the cycle (Figure 1-H0 to H22, and Additional file 1), at H0 post infection (H0 p.i.) and H2 p.i., internalized bacteria are detectable in the amoebal cytoplasm, appearing as red-stained coccoïd shapes with Gimenez staining and extremely bright stained forms with DAPI labeling. Then, from H4, similar to a viral replication cycle, we observed an eclipse phase, with complete disappearance of the small coccoid forms in the cytoplasm. Instead, the growth of large dense forms, purple stained in Gimenez and brightly marked with DAPI, was observed at this stage. These large, dense bodies grow in the cytoplasm from H6 p.i. to H20 p.i. Electron microscopy allows us to more precisely observe the unique multiplication mode of *B. massiliensis*. At H0 and H2 after infection, internalized bacteria were observed in the cytoplasm of amoebae included in a vacuole. At H4, H6, H8, bacteria start to lose their electron-density and progressively increase in size from 1.2 μm +/− 0.4 μm length and 0.8 μm +/− 0.3 μm width to 1.5 μm +/− 0.4 μm length and 1.1 μm +/−0.3 μm width. Between H8 and H12, bacterial bodies continue to grow, reaching 3 μm +/− 0.7 length and 2 μm +/− 0.6 width (see Additional file 2), and forming large accumulations of amorphous material. These structures appear to grow in one unique vacuole in the cytoplasm of the amoeba. Besides the growth of these amorphous bodies, at H15 p.i. we observed the appearance of internal membranes which seem to be precursors to mature bacteria (Additional file 3). After the appearance of these membranes, the amorphous material starts to differentiate into polylobulated structures. These structures then separate into extremely long bacillary forms. These growing larger forms progressively invade the entire amoeba cell. At H20, the long bacillary forms split into numerous, apparently unstructured small bacterial forms. At H22 p.i., the immature small forms reach high density and mature bacteria (both crescent and round forms) accumulate, filling the cell cytoplasm.

### Quantification of bacterial multiplication and effect on amoebae

The quantity of *B. massiliensis* DNA increased steadily to reach a plateau at H24 (see Additional file 4, part A). Another increase was observed around H30 and might reflect re-infection of uninfected amoeba still present in the culture (Not shown). The infectious bacteria count, measured by end-point dilution, did not increase steadily, in contrast to the steady increase of the DNA amount. An abrupt increase of infectivity was observed between H20 and H25 when mature particles are released as observed by electron microscopy, when cell lysis occurs. Comparative analysis of replication cycles showed that *B. massiliensis* grew faster and to greater quantities compared to *Legionella drancourtii*: between H0 and H30, quantitative PCR showed an increase of 5 log of the amount of *B. massiliensis* DNA, whereas we observed an increase of only 1.5 log of the amount of *L. drancourtii* DNA. Based on DNA production, we evaluated the doubling time of *L. drancourtii* and *B. massiliensis* at approximately 300 and 150 min, respectively [16].

Infection with *B. massiliensis* led to a nearly complete lysis of the amoeba within 70 hours. After 70 hours of culture, the amount of infected amoeba decreased by approximately 85%, whereas a non infected culture of *A. polyphaga*, used as a negative control, showed no detectable loss of amoeba over the same experiment duration (see Additional file 4, part B). To further assess the pathogenicity of *B. massiliensis* in amoeba, we compared the pathogenic effect of *B. massiliensis* with that of *L. drancourtii*, (see Additional file 4, part C). The experiments conducted with *L. drancourtii* were done using amoeba of the species *A. castellanii*, because *L. drancourtii* does not grow well in *A. polyphaga,* whereas *B. massiliensis* shows the same pathogenicity and growth rate in both amoeba. Indeed, no reduction of amoeba at H24 was observed with *B. massiliensis* grown in *A. castellanii*, consistent with the *A. polyphaga* results. In contrast, with *L. drancourtii,* the count of amoeba dropped by more than 50%*.* The negative control showed a natural loss of ~40% of *A. castellanii* in 24 h in the non-nutritive PAS buffer (see Additional file 4, part D), suggesting that *L. drancourtii* has a pathogenic effect on *A. polyphaga*, whereas *B. massiliensis* might have an early protective effect on amoeba, at least during the first 48 h of infection, followed by a pathogenic effect. Indeed, we found that at H70, the drop in the living amoeba count with *B. massiliensis* was 85% although, with the more severe pathogen *L. drancourtii,* there was no living amoeba left at the same time post-infection.

### Genome sequencing and general features of the *B. massiliensis* genome

Amplification and sequencing of the complete 16S rRNA gene led to a 1501 bp long sequence, which was deposited in Genbank (GQ495224). A comparison of *B. massiliensis* 16S rRNA with those available in the NR database strongly suggests that it belongs to a recently proposed bacterial phylum that currently includes only one published draft genome, JCVI TM6SC1 of Candidatus phylum TM6 [13], but is additionally represented by numerous related sequences from diverse environments. The 16S RNA sequence of *B. massiliensis* is 95% identical to the JCVI TM6SC1 sequence whereas the best match is ~96% identity with a 16S rRNA sequenced from an acidic cave wall biofilm [17]. Complete genome sequencing was performed after these findings.

**Figure 2:**
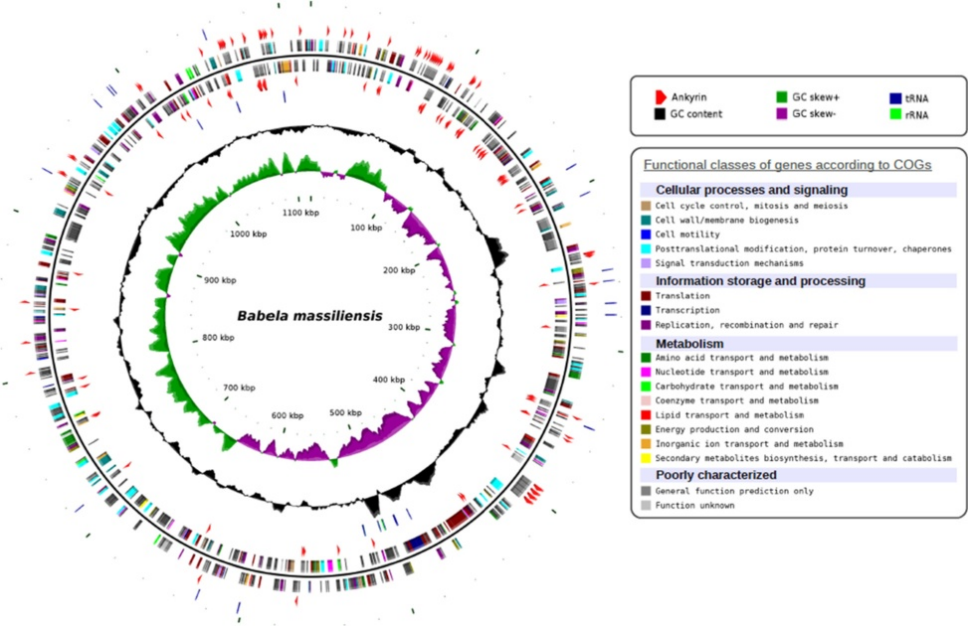
**Circular representation of the** ***Babela massiliensis*** **chromosome.** Circles from the center to the outside: GC skew (green/purple), GC content (black), tRNA (blue) and rRNA (green) on forward strand, ankyrins (red arrows) on forward strand, genes on forward strand colored by COGs categories, genes on reverse strand colored by COGs categories, ankyrins (red arrows) on reverse strand.

The genome of *B. massiliensis* consists of a single circular chromosome with the size of 1,118,422 bp and a GC content of 27.4%. In total, 981 protein-coding sequences (CDS) were identified, with 198 (~20%) of the predicted proteins showing no detectable sequence similarity to other proteins in public databases (including those of JCVI TM6SC1) (Figure 2). Notably, the *B. massiliensis* genome encompasses a relatively large number of tandem gene duplications (37, compared to 17 in TM6SC1). The CDS annotation shows a relatively low coverage with Clusters of Orthologous Genes (COGs) (652 genes, i.e. 66% of the CDS, compared to the average of approximately 75% for a representative genome set) [18]. The genome encodes one typical rRNA operon, 38 tRNAs (for all amino acids), 4.8S (Signal Recognition Particle) RNA, tmRNA and the RNA component of RNAse P. No phage or plasmid- related genes were identified, and only one IS4 family transposase gene was detected. This observation is in a sharp contrast to the high proportion (24% of the protein-coding genes) of predicted mobile genetic elements in the genome of “Ca. *Amoebophilus asiaticus*”, another intracellular parasite of amoeba [19]. As expected of an obligate intracellular symbiont, *B. massiliensis* encodes only 5 predicted transcription regulators. A single putative replication origin was predicted at 152 nt upstream of the start of the *dnaA* gene using GCskew analysis.

### Phylogenetic analysis and taxonomic affinities of *B. massiliensis* genes

**Figure 3:**
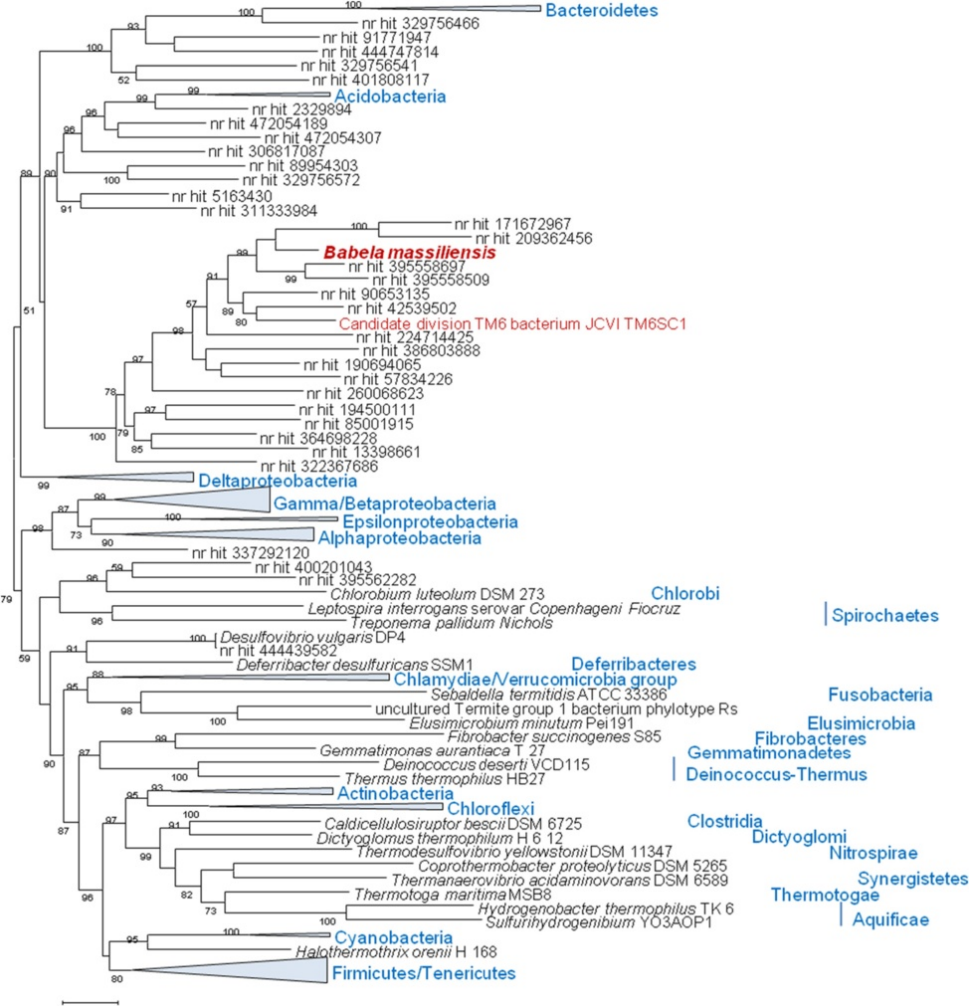
**Maximum-Likelihood tree of 16S RNA gene constructed with TreeFinder.** Environmental sequences that are currently not assigned to a particular bacterial species [nr (NCBI non-redundant nucleotide sequence database) hits] are identified by Gene Identification (GI) numbers. Multiple members of strongly supported, monophyletic bacterial phyla are collapsed and shown by triangles, with the names of the phyla rendered in blue. The bootstrap support values for each internal branch are given as percentage points.

**Figure 4:**
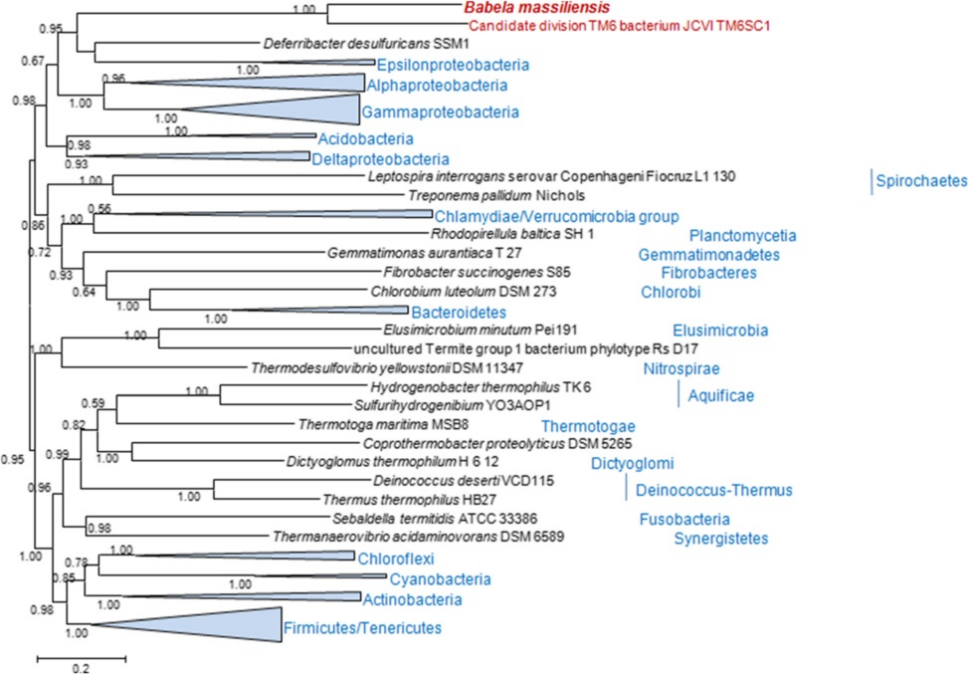
**Maximum-Likelihood tree of ribosomal proteins.** A concatenated alignment of 50 ubiquitous bacterial r-proteins was used to reconstruct the tree (6,092-positions). Bootstrap support values are given on the 0 to 1 scale. Other designations are as in Figure 3.

The phylogenetic tree of 16S rRNA built for a representative set of the bacterial sequences available in the NR database confidently groups *B. massiliensis* with JCVI TM6SC1 and numerous other uncultivated environmental bacteria. Together, these sequences comprise a clade that clusters with Acidobacteria (Figure 3); this latter grouping, however, was weakly supported by bootstrap. In the maximum likelihood phylogenetic tree of concatenated ribosomal proteins for a selected set of bacteria covering all the major bacterial lineages (Figure 4) [20], the TM6-*Babela* clade belongs to a major branch that includes all Proteobacteria along with Acidobacteria and Deferribacter, bacterial groups that traditionally are not grouped together. The results of our phylogenetic analysis are generally compatible with those reported for JCVI TM6SC1 where the AMPHORA2 method using 29 protein phylogenetic markers placed JCVI TM6SC1 with Acidobacteria [13]. Taken together, the results of phylogenetic analysis suggest that *B. massiliensis*, JCVI TM6SC1 and their numerous uncharacterized relatives identified among environmental sequences comprise a distinct phylum within a putative “extended Proteobacteria” superphylum.

In a sharp contrast with the clear-cut results of the phylogenetic analysis of universal proteins, the taxonomic distribution of the best BLAST hits (see Additional file 5, part A) and the results of phylogenomic analysis of conserved protein families (see Additional file 6) suggest diverse affiliations for the protein-coding genes of *B. massiliensis*. Unexpectedly, given the high sequence similarity between rRNA sequences and the sequences of the universally conserved proteins, predicted proteins of *B. massiliensis* produce only 470 bidirectional best hits with JCVI TM6SC1 (48% of the CDS). Thus, there is a substantial difference in the gene content between these organisms that is likely to result from differential gene loss and acquisition of genes from different sources, in turn determined by the drastically different lifestyles of the two bacteria. Notably, the GC content of *B. massiliensis* also significantly differs from that of JCVI TM6SC1 (27% vs 36%).

Those genes of *B. massiliensis* that have apparent orthologs (bidirectional best hits) in JCVI TM6SC1 show a small excess of genes with an apparent affinity to Deltaproteobacteria and Clostridia. In contrast, those proteins that are either missing in JCVI TM6SC1 or are more similar to homologs from other organisms than to those from JCVI TM6SC1 show some excess of best hits to homologs from Bacteroidetes-Chlorobi, Chlamydiae/Verrucomicrobia and eukaryotes (see Additional file 5, part A). Interestingly, the bacterial phyla with an excess of *Babela*-specific best hits include several obligate symbionts of amoeba. Together with the excess of eukaryotic homologs, these observations are compatible with extensive HGT between parasites (symbionts) and between parasites and the host within amoeba, in agreement with the previous observations on amoeba as a “melting pot” of HGT and notwithstanding the obvious caveats associated with the use of sequence similarity as an indicator of evolutionary relationships [10,12,21-23].

The results of an automated phylogenomic analysis of 165 conserved protein families also suggest a scatter of the phylogenetic affinities of *Babela* among diverse bacterial taxa. Control analysis with several other bacteria indicates that this approach is generally capable of correctly assigning bacteria to their respective phyla (see Additional files 6 and 7). The scatter of the apparent phylogenetic affinities of *Babela* genes most likely results, first, from the lack of sequences from members of the same putative phylum (other than JCVI TM6SC1) in the current databases, which results in increased incidence of phylogenetic artifacts, and second, from actual acquisition of genes from various sources via HGT. It is difficult to distinguish one source of the observed phylogenetic diversity from the other but the clear differences in the apparent gene origins between *Babela* and JCVI TM6SC1, in particular the smaller number of eukaryotic homologs in the latter (Additional file 5, part B), suggest that the contribution of the lifestyle-dependent HGT is substantial. Examples of apparent different origins of the orthologs in *Babela* and TM6 are given in the Supplemental Information (Additional file 8).

### Phylum-specific and lineage-specific genes of *B. massiliensis*

A comparison of the protein-coding genes of *B. massiliensis* to those of bacteria with similar genome sizes revealed 33 *B. massiliensis* genes that are absent in other genomes from this set (Additional file 9); 18 of these genes are also present in JCVI TM6SC1 suggesting an early acquisition. This group includes several genes involved in Tfp pilus system assembly, NurA 5'-3' nuclease, eukaryotic-type DNA primase and a leucine-rich repeat protein. Among the genes that are present in *B. massiliensis* but not in JCVI TM6SC1 are several genes that are common in archaea and eukaryotes but are rarely found in mesophilic bacteria including peptide chain release factor 1 (eRF1), termostable 8-oxoguanine DNA glycosylase, and a protein of the archease family that is implicated in tRNA splicing in archaea and eukaryotes.

### Genome reduction and limited metabolic capabilities of *B. massiliensis*

*B. massiliensis* and JCVI TM6SC1 have similar genome sizes indicative of reduced gene complements consistent with the symbiotic (parasitic) lifestyle given that so far all bacteria with fewer than 1200 protein-coding genes are obligate or facultative symbionts or parasites [18]. The absence of the genes for the enzymes of central metabolic pathways is a good indicator of genome reduction associated with symbiotic or parasitic lifestyles. The analysis of the relevant gene sets (Additional file 10) suggests that neither *B. massiliensis* nor JCVI TM6SC1 are able to synthetize amino acids, nucleotides, cofactors, fatty acids and isoprenoids, and therefore depend on their hosts as the source of the great majority of metabolites. Among the few exceptions, there are several enzymes of glycolysis, phospholipid biosynthesis and thymidylate biosynthesis (Additional file 10). Unlike JCVI TM6SC1, *B. massiliensis* does not encode murein biosynthesis pathway genes and accordingly is predicted to be unable to produce the peptidoglycan cell wall. Compared to other bacteria with 800 to 1100 protein-coding genes (47 genomes extracted from the current genomic databases), both organisms follow the common trend of genome reduction (Additional file 10), consistent with the recent observations on the convergence of the genome reduction processes, especially with regard to central metabolic pathways, among numerous intracellular symbionts [24]. Among the genes that were probably lost in the common ancestor of JCVI TM6SC1 and *B. massiliensis* but are present in most (37 to 47) of the small bacterial genomes (Additional file 11), there are several genes for glycolytic enzymes and several genes encoding components of information-processing systems. The latter group of genes includes the delta subunit of DNA polymerase III, 16S RNA methyltransferase RsmD, NAD-dependent DNA ligase (replaced by ATP-dependent DNA ligase), and RNAse III. Also notable is the absence of methionyl-tRNA formyltransferase and N-formylmethionyl-tRNA deformylase which implies a distinct mechanism of translation initiation involving initiator Met-tRNA similar to the mechanism identified in archaea and several bacteria, mostly symbionts as well [25]. Apparent losses of generally conserved genes that are specific for *B. massiliensis* include the FEN1-family 5'-3' exonuclease, tRNA-dihydrouridine synthase, and two genes for enzymes that catalyze the final steps of pyrimidine synthesis, uridylate kinase and CTP synthase, as well as several genes involved in cell division (see below).

### Radical reduction of the cell division machinery

Given the unique cell multiplication mechanism discovered in *B. massiliensis* (see above), we examined in greater detail the genes implicated in cell division. Compared with JCVI TM6SC1, *B. massiliensis* lacks many important genes involved in cell division such as MreB, MreC, FtsB, FtsK, FtsW, FtsI as well as Smc, the ATPase involved in chromosome segregation. The remaining division-related genes that are shared with JCVI TM6SC1 include FtsZ, the tubulin-related self-assembling GTPase and the structural component of the Z-ring, which forms in the cell midplane and serves as the scaffold for the divisome assembly; FtsA, the actin-related ATPase essential for Z-ring formation; and ZapA,B, two proteins that facilitate the Z-ring assembly and stabilize it *in vivo* [26]. The absence of the genes for cell envelope biosynthesis suggests that *B. massiliensis* lacks a cell wall. Furthermore, the absence of MreB and MreC components that are responsible for the rod shape formation in bacteria is compatible with the coccoidal shape of *B. massiliensis* observed in this work [27]. The deep reduction of the cell division apparatus that was observed in *B. massiliensis* is not unusual for symbiotic bacteria with extremely small genomes [26] but was unexpected in an organism with nearly 1000 genes and much more pronounced than in the related JCVI TM6SC1 bacterium. Beyond this drastic reduction, we did not detect any unique genes that could be implicated in the unusual cell multiplication mechanism. It remains a possibility that this mechanism involves still uncharacterized bacterial genes and/or molecular machinery of the host amoeba.

To assess the functioning of the cell division machinery, RT-PCR assays for the *ftsZ*, *ftsA*, and *groEL* (employed as a control) genes were performed with both *B. massiliensis* and *L. drancourtii,* in order to compare *B. massiliensis*, with its unique multiplication mechanism, to an amoeba-infecting bacterium with a typical division process. As shown in Additional file 12 (Additional file 12, part A), expression of *ftsA* and *GroEL* was detected throughout the replication cycle whereas *ftsZ*, which encodes the major component of the Z ring, was expressed only at the end of the replication cycle of *B. massiliensis*, starting from H16 p.i. Additional file 10 also shows that expression of *ftsZ* occurs at the beginning of the division cycle of *L. drancourtii*, in contrast to *B. massiliensis* (Additional file 12, part B)*.* This difference in the patterns of *ftsZ* expression is likely to reflect the unique mechanisms of cell multiplication in *B. massiliensis* whereby the bacterium grows to form large bodies that split into individual bacterial cells at a late stage in the replication cycle.

### Genomic signatures of an intracellular pathogen

Intracellular symbionts often engage in a mutualistic relationship with the host by providing metabolites that the host cannot synthesize, in particular, amino acids [27]. However, given the apparent absence of any complete metabolic pathways, it is unlikely that *B. massiliensis* employs this strategy, in agreement with the observed lysis of the infected amoeba, which suggests that *B. massiliensis* is a genuine pathogen rather than a symbiont (see above). Nevertheless, it cannot be ruled out that *B. massiliensis* provides some intermediates to the host. For example, it remains unclear what could be the functions of *B. massiliensis* enzymes that catalyze intermediate reactions in several pathways, such as (acyl-carrier-protein) S-malonyltransferase (FabD), branched-chain amino acid aminotransferase (IlvE) or ATP sulfurylase (sulfate adenylyltransferase). The same applies to detoxification pathways given that *B. massiliensis* encodes several hydrolases of different families, including four NUDIX family hydrolases, and two SodA-like superoxide dismutases that could be involved in cell “house cleaning” [28].

As expected and in contrast to the paucity of metabolic enzymes, *B. massiliensis* encompasses numerous genes for proteins that are implicated in the transport of most essential nutrients into bacterial cells. Like many intracellular symbionts, *B. massiliensis* encodes three ATP/ADP translocases for ATP import, mitochondrial carrier family transporters with unknown specificity, three porins, a nucleoside permease of the ABC transporter family, Mg/Co/Ni transporter (MgtE) and several putative proton channels, including MscS and MhpC, several Na + antiporters, and other transporters. There is also a plethora of predicted peptidases, both extracellular and intracellular, including Dcp-like Zn-dependent oligopeptidase, PqqL-like Zn-dependent peptidase, two clostripain-like C11 family, peptidase, PepB-like leucyl aminopeptidase, PepP-like Xaa-Pro aminopeptidase, periplasmic serine protease SppA, and periplasmic protease Prc.

**Figure 5:**
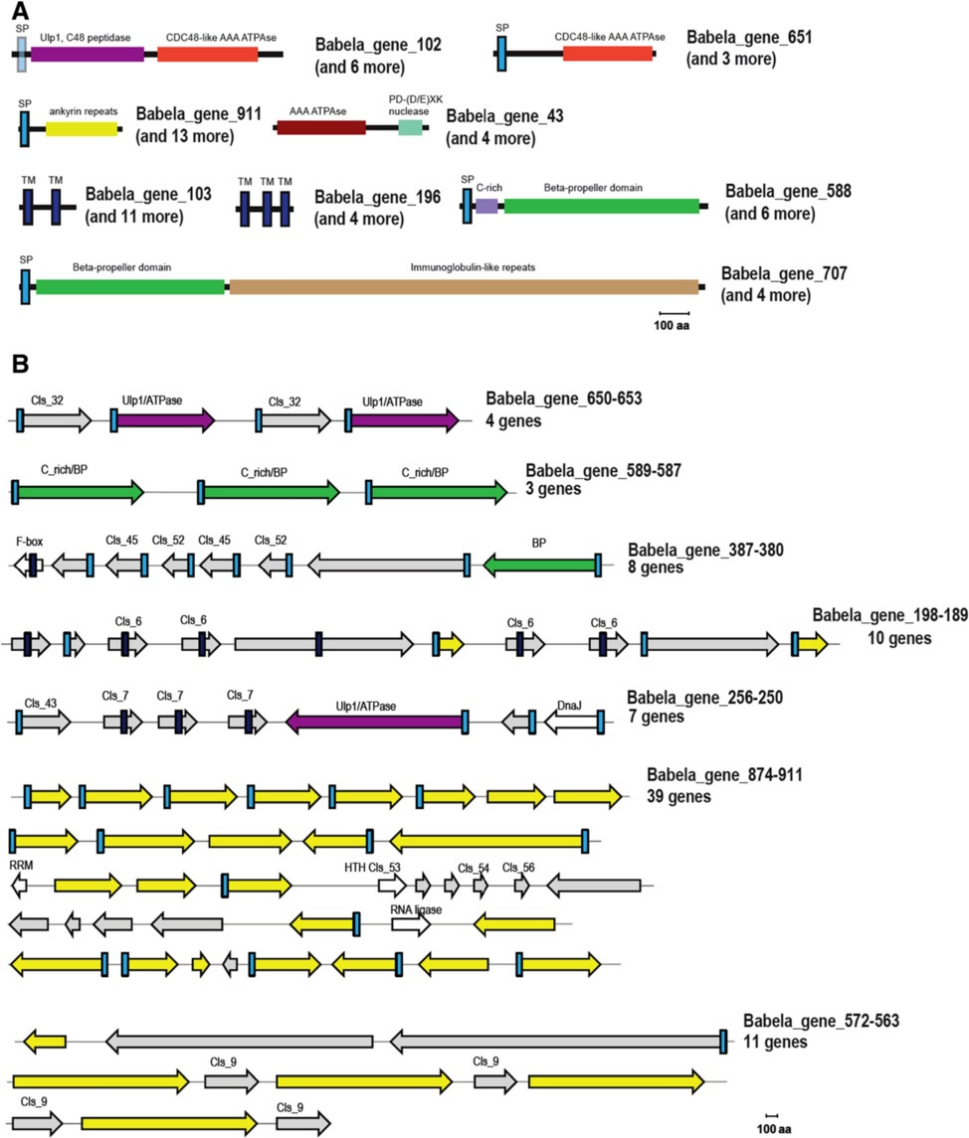
**Unique genomic features of** ***B. massiliensis.*** **A**. Domain organization of selected proteins from families expanded in *B. massiliensis*. The proteins are shown roughly to scale. The identified domains are shown by block colored shapes. Homologous domains are shown by the same color. **B**. Genomic islands encoding putative host interaction genes. The genes are shown by block arrows, roughly to scale. Color coding is the same as in **A**. Proteins specific for *B. massiliensis* in which no known domains were identified are shown in gray; protein implicated in housekeeping functions are shown by outline arrows. For the protein families expanded in *B. massiliensis* the respective cluster number (eg. Cls_7) is indicated. Abbreviations: TM – transmembrane helix, SP – signal peptide, BP – beta propeller; PD –DExK is a conserved motif in the respective nuclease family.

Despite its small genome size, *B. massiliensis* encodes a full complement of proteins with chaperone-like functions, and moreover, shows expansion of several families such as ATP-dependent Zn proteases and thioredoxin reductases. In addition, there are three DnaJ-domain-containing proteins that are specific for *B. massiliensis* (Figure 5). The repertoire of chaperones encoded by *B. massiliensis* is notably larger than those in most intracellular bacteria including parasites of amoeba (Figure 5 and Additional file 11). The biological underpinning of this excess of chaperones is unclear although it is tempting to link it tentatively with the unusual cell division process.

### Similar adaptive traits between *B. massiliensis* and other amoeba-associated bacteria and viruses

**Figure 6:**
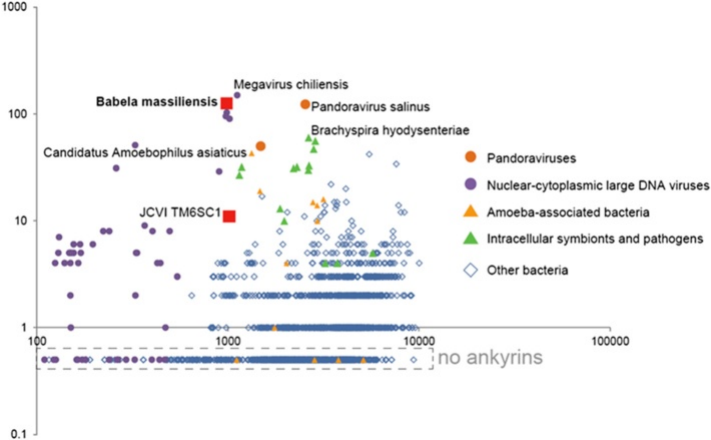
**Distribution of the number of ankyrin repeat-containing proteins in complete bacterial genomes.** X-axis, number of protein coding gene in a genome; Y-axis, number of ankyrin repeat-containing proteins in a genome.

Genomic analysis of amoeba-associated bacteria revealed several protein families that are often present specifically in these genomes [19] and, interestingly, also in the genomes of some of the giant viruses infecting amoeba. *B. massiliensis* encompasses many of these families. Most notably, all these bacteria and viruses encode numerous ankyrin repeat-containing proteins that are involved in a wide range of protein-protein interactions and are thought to function as modulators of various host protein activities and post-translational modifications, promoting virulence and persistence of bacteria within the host [29-32]. Many viruses with large genomes, in particular representatives of the Nucleo-Cytoplasmic Large DNA Viruses (NCLDV; proposed order Megavirales) of eukaryotes [33], also encode multiple ankyrin repeats implicated in virus-host interactions [34]. *B. massiliensis* encodes 126 ankyrin repeat-containing proteins, by far the largest number among the sequenced bacterial genomes. The majority of these proteins (n = 79) contain a predicted signal peptide (see Additional file 9) and accordingly, are most likely secreted into the cytoplasm of the infected amoeba. Notably, the second largest number of ankyrin repeat-containing proteins (n = 94) was detected in Candidatus *Amoebophilus asiaticus*, another amoeba pathogen (Figure 6) [19].

*B. massiliensis* also encodes many other “eukaryotic” proteins most of which contain different types of repeat modules and are involved in protein-protein interactions and signal transduction including several WD40 repeat-containing proteins and many other beta-propeller and Ig-like domain containing proteins (Figure 5), a leucine-rich repeat (LRR) protein, and a TPR repeat protein. Some of these proteins are predicted to be secreted and, analogous to the ankyrins, might interfere with host cellular processes and/or restructure the intracellular environment to facilitate bacterial reproduction.

In parallel with intracellular symbionts and giant viruses, particularly those associated with amoeba, genome analysis clearly shows that *B. massiliensis* exploits the host ubiquitin system. Indeed, it encodes an F-box containing protein which, as shown for viruses, can interact with cullin based ubiquitin ligases [34,35], a RING finger domain protein, which is a potential E3 component of ubiquitin ligase, and three secreted ubiquitin C-terminal hydrolases of the C19 peptidase family [36]. In addition, among the protein families that are expanded in *B. massiliensis* (Figure 4 and Additional file 9) we identified 9 closely related and specific to this bacterium proteins containing an AAA ATPase domain and a derived, circularly permuted homolog of the eukaryotic desumoylating Ulp1 peptidase of the C48 family (Figure 5, Additional file 13). These proteins also contain predicted signal peptides (albeit somewhat atypical ones) and so at least some of them are likely to be secreted, compatible with a function in the cytoplasm of the amoeba. In several members of this family, the peptidase domain appears to be inactivated. At present, this domain fusion and the permuted form of the Ulp1-like peptidase are unique to *B. massiliensis*. In eukaryotes, the homologous peptidases are involved in SUMO maturation and deconjugation of SUMO from targeted proteins, contributing to the regulation of a diverse range of biological responses including cell division and signal transduction [37-39]. Recently, it has been shown that viruses interact with components of the sumoylation pathway through a variety of mechanisms [40]. Conceivably, some intracellular bacteria including *B. massiliensis* can similarly affect the host cell cycle.

Like many pathogenic bacteria, *B. massiliensis* can be predicted to employ a type II secretion system (the only secretion system detected in the genome) for export of proteins involved in parasite-host interactions [31]; a homologous system is also present in JCVI TM6SC1.

### Potential unique components of host adaptation systems

Further analysis of expanded protein families in *B. massiliensis* suggests that, in addition to mechanisms that are common to diverse intracellular parasites and symbionts, it might employ other, previously undetected mechanisms of interaction with the host. For example, we identified a family of predicted membrane proteins that are mostly found in the neighborhood of the aforementioned proteins containing the Ulp1-like desumoylation domain (Figure 5) suggestive of a functional link between these two families. Another family includes 7 large secreted proteins that consist of a cysteine-rich N-terminal domain followed by a beta-barrel domain, the domain architecture reminiscent of animal low density lipoprotein receptors (Figure 5) [41,42]. There are many more predicted secreted and membrane-associated proteins encoded in the *B. massiliensis* genome, several of which are encoded within genome islands enriched in ankyrins, suggesting that all these proteins are involved in distinct parasite-host interactions (Figure 5).

Unexpectedly, *B. massiliensis* was found to encode two toxin-antitoxin pairs, namely HicA-HicB and MNT-HEPN. These systems might be involved in a persistence mechanism that so far is not known to exist in amoebal symbionts and more generally in bacteria with similar genome sizes [43].

## Discussion

During the last few years, the typical process of discovery of new prokaryotes and even prokaryotic phyla has dramatically changed. It has become common practice to discover microbes through metagenomics so that a (nearly) complete genome representing a group of bacteria or archaea becomes available before any biology of the respective organisms is known. This was the case with the recent description of a putative phylum typified by JCVI TM6SC1 [13] .With the serendipitous discovery of *Babela massiliensis,* there is now a cultured (albeit only in amoeba) representative of this potential phylum with a closed genome that is amenable to study with at least some of the traditional microbiological methods. The existence of numerous related sequences in metagenomes isolated from diverse environments indicates that the TM6 phylum is widespread and versatile. Phylogenetic analysis described here suggests that this phylum might belong to a superphylum together with Acidobacteria, Deferribacter and Proteobacteria.

Despite the high sequence similarity between the conserved genes of *B. massiliensis* and JCVI TM6SC1, the gene repertoires of the two bacteria differ dramatically, with the gene set of *B. massiliensis* showing multiple signatures of the intra-amoebal life style. Free-living amoebae are unicellular phagocytic protozoa that are widely represented in the environment and appear to be melting pots of gene exchange between intracellular parasitic and symbiotic bacteria and giant viruses [12]. Phylogenomic analysis suggests diverse origins for *B. massiliensis* genes although caution is due in the interpretation of these observations, given the distant relationship between the putative TM6 phylum and other bacteria.

*B. massiliensis* appears to be an obligate intracellular parasite; indeed, all attempts to cultivate it outside amoeba have failed, despite using several liquid and solid media under a variety of conditions. The genome of *B. massiliensis* is relatively small, approximately 1.2 Mb, and shows many of the signatures of genome reduction that is a characteristic of strictly intracellular microbes [44]. In particular, *B. massiliensis* has lost effectively all metabolic pathways so that the bacterium depends on the amoebal host for (nearly) all metabolites for which it encompasses a versatile set of transporters.

*B. massiliensis* multiplies via a highly unusual, budding mechanism. Genes for many of the typical components of the bacterial cell division machinery are missing although FtsZ, the key protein involved in septation, is present. Genome analysis suggests that *B. massiliensis* lacks a peptidoglycan cell wall. Thus, it seems likely that budding of *B. massiliensis* is, at least in part, a non-enzymatic, mechanical process, analogous in that respect to the division of bacterial L-forms [45,46]. This process could be regulated by excessive membrane phospholipid production [46], and it might be relevant that *B. massiliensis* retains several enzymes of phospholipid biosynthesis.

The extensive genome reduction notwithstanding, *B. massiliensis* encodes numerous proteins that are implicated in various facets of parasite-host interaction. A notable aspect of this apparent adaptation is the extensive proliferation of ankyrin repeat-containing proteins. Ankyrin repeats are common among intracellular pathogenic bacteria, e.g. *L. pneumophila, Anaplasma phagocytophilum, Coxiella burnetii, Rickettsia* sp., and *Orientia tsutsugamuchi* [32], in particular those infecting amoeba. Notably, the pathogenicity of *Coxiella burnetti* has been shown to depend on the ankyrin repeats [47]. In parallel, similar observations have been reported for large viruses, e.g. Myxoma virus, a member of the family Poxviridae, in which the ankyrin repeat proteins are required for abrogation of the host defense and accordingly for virulence [48]. Among all bacteria and viruses sequenced so far, *B. massiliensis* shows the highest content of ankyrin repeats when normalized by genome size, suggesting the importance of these proteins in the interaction with the amoeba. In addition to the ankyrin repeats, *B. massiliensis* is rich in other “eukaryotic” proteins containing repetitive domains involved in protein-protein interaction. Many of these proteins are predicted to be secreted and accordingly are directly implicated in the interaction between the parasite and amoebal proteins.

Perhaps the most striking feature of the inferred adaptations of *B. massiliensis* to the intracellular lifestyle is the prominence of proteins related to the ubiquitin signaling system. Although other intracellular bacteria and giant viruses also encode proteins predicted to interfere with ubiquitin signaling, the number and diversity of such proteins in *B. massiliensis* is without precedence. In particular, the family of 7 paralogous genes that encode derived homologs of desumoylating enzymes fused with ATPase domains is so far unique to this bacterium.

The discovery and genome sequencing of *B. massiliensis* open up at least three research directions. First, this bacterium is currently the most promising model to study the biology of an apparently widespread and diverse but effectively uncharacterized putative bacterial phylum. Second, understanding the mechanisms of cell budding in *B. massiliensis* could shed light on general aspects of the evolution of cell division. Finally, of considerable interest is the characterization of the pathogen-host interaction in the *B. massiliensis*-amoeba system, in particular the impact of the bacterium on ubiquitin signaling.

## Conclusions

*Babela massiliensis* currently is the only representative of the candidate TM6 phylum that can be grown in the laboratory and for which the complete genome sequence was determined. The genome analysis of this obligate intracellular parasite of *Acanthamoeba* sp. shows striking divergence of the gene repertoire from that of JCVI TM6SC1, the only other available draft genome sequence from the same phylum, conceivably due to different lifestyles. Phylogenomic analysis suggests that *B. massiliensis* acquired multiple genes by horizontal transfer from diverse sources including other bacteria and eukaryotes, conceivably, as a consequence of the sympatric lifestyle within the amoebal cytoplasm. Numerous genes of *B. massiliensis* are implicated in specific interactions with the amoeba host including the record number of ankyrin repeat proteins and diverse proteins predicted to affect the ubiquitin system. A striking biological feature of *B. massiliensis* is its unprecedented cell division mode which involves initial formation of large polymorphic bodies that fill the cytoplasm of the infected amoeba and subsequently rapidly split into numerous coccoid bacterial cells. This unique mechanism of cell division is associated with the loss of numerous components of the cell division machinery and delayed expression of the *ftsZ* gene, the key component of the Z ring that is essential for septation. Further study of *B. massiliensis* is expected to yield insights into bacterial cell division, the biology of parasite-host interaction and the characteristics of the emerging TM6 phylum of bacteria. This discovery may be regarded as one more illustration of the renaissance of microbial culture recently emphasized by the culturomics concept [49].

## Methods

### Isolation and morphological characterization of *Babela massiliensis*

Two liters of water were collected in a cooling-tower located in Paris, filtrated through a 0.22 μm pore-sized filter and these filters were shaken in 2 ml of sterile Page’s amoebal saline (PAS). Sample was inoculated onto an amoebal monolayer of the species *Acanthamoeba polyphaga* and *Babela massiliensis* was isolated and further cultured using an amoebal co-culture procedure, as described previously [50]. Details regarding isolation, morphological characterization, pathogenicity for amoeba and preliminary molecular identification are provided in Additional file 14. The first isolated strain, BaBL-1, was deposited in the bacterial collection CSUR (Collection de Souches de l’Unité des Rickettsies, Marseille France) under the collection number CSUR P554.

### Replication cycle of *B. massiliensis*

A co-culture was prepared by inoculating *A. polyphaga* rinsed in PAS with a *B. massiliensis* suspension in two 75 ml culture flasks at 32°C. After 2 hours of incubation, the amoeba monolayer was washed 3 times in PAS buffer in order to eliminate not internalized bacteria. This time was considered as H0. The two cultures flasks were pooled after resuspending infected amoeba, and 10 ml distributed into 12 new culture flasks. A culture flask containing only amoeba was used as negative control. At H0, H2, H4, H6, H8, H10, H12, H14, H16, H18, H20 and H22, one flask was used: two slides were prepared by cytocentrifugation of 200 μl culture, for Gimenez staining and DAPI nucleic acids labeling (Molecular probes). The remaining of the 10 ml of co-culture were centrifuged at 2000 rpm during 10 minutes and the pellets with infected amoeba were fixed in a 2,5% glutaraldehyde solution and stained for transmission electron microscopy study. Measure of bacterial growth was performed both by Real-time PCR assays and bacterial particles counting by end-point dilution method. Functioning of the cell division machinery was measured with RT-PCR assays for the *ftsZ*, *ftsA*, and *groEL* (employed as a control) genes, for both bacteria *B. massiliensis* and *L. drancourtii.* Details methods for Real-time PCR assays and Reverse Transcription PCR assays are provided in Additional file 14.

### Sequencing of *B. massiliensis* genome

Detailed methods for presumptive molecular identification are provided in Additional file 14*.* For whole genome sequencing, bacteria were inoculated into 100 culture flasks containing 50 ml PYG and 5 ml *A. polyphaga*. After multiplication and complete lysis of the amoeba, culture supernatant was centrifuged at 2000 rpm for 10 minutes and filtrated through 5 μ pore size filter, to eliminate amoebal debris. Supernatant was then centrifuged at 5500 rpm for 30 minutes and washed three times in PAS. The pellet was resuspended in 50 ml PAS, and purified through 25% sucrose solution. The pellet was resuspended in 40 ml sterile PBS. Total DNA was extracted using phenol-chloroform and prepared for sequencing as previously described [51]. The genome of *B. massiliensis* was sequenced on the 454 Roche GS20 and 454-Roche GS FLX Titanium [52]. A series of shotgun sequencings was performed, in addition of the pyrosequencing of a 3 kb paired-end libraries. The paired-end library was constructed according to the 454 Titanium paired-end protocol proposed by Roche. The 454 sequencing generated 397,090 reads (67,39 Mb) assembled into contigs and scaffolds using Newbler version 2.7 (Roche) and Mira 3.4 [53]. SSPACE software v1.0 [54] combined to GapFiller V1.10 [55] were also used to enhance the assembly. In addition, a pyrosequencing with the SOLiD platform version 4 (Life Technologies) completed the sequence determination of *Babela massiliensis*. This run produced 5,054,896 usable reads from a paired-end library fragments of 235 bp long (150 bp for inserts and 50/35 bp for reads). This SOLiD data helped to check and improve the quality of the main assembly, which were combined thanks to CLC Genomics Workbench v4.7.2 software (CLC bio, Aarhus, Denmark). Finally, leaving gaps or uncertainties were achieved using some polymerase chain reaction (PCR) amplifications and sequencings with specifically-designed primers. The complete, fully annotated genome was deposited in the EBI database under the accession number HG793133.

### Sequence data for genome comparison and phylogenetic analysis

For comparative genomics and phylogenetic reconstruction based on amino acid sequences, we used 2262 completely sequence bacterial and archaeal genomes available in Refseq database [20] (as of February 2013). For 16S phylogenetic reconstruction, 16S RNA sequences of the 110 representative bacterial genomes used for conserved ribosomal protein tree construction was taken. Additionally, the BLASTN program [56] was run against the NCBI NR database using *B. massiliensis* 16S RNA as a query. The 1,000 top hits were taken, and complete genomes were removed from the list; the remaining 986 sequences were clustered using the BLASTCLUST program (http://toolkit.tuebingen.mpg.de/blastclust) with a 97% identity cutoff. This procedure resulted in 40 sequences representing closest homologs of *B. massiliensis* 16S RNA gene, which were added to the 16S sequence pool, together with JCVI TM6SC1 16S RNA sequence.

### Genome annotation and sequence analysis

The protein-coding genes in the *B. massiliensis* genome were predicted using the GeneMarkS software [57]. Translated protein sequences (ORFs) were searched against the NCBI Refseq protein sequence database and separately against the JCVI TM6SC1 protein set using BLASTP [55], with an e-value cutoff of 0.01. Conserved domains were identified by searching the Conserved Domain Database (CDD version 3.10) [58] using the RPS-BLAST [59]. *B. massiliensis* and JCVI TM6SC1 proteins were assigned to Clusters of Orthologous Groups of proteins (COGs) with PSI-BLAST [56], using position-specific scoring matrices derived from multiple alignments of protein sequences in 4,738 COG [60]. Top Refseq hits, identified conserved domains and COG annotations, combined with extensive manual curation, were used for annotation of *B. massiliensis* proteins. The t-RNA genes were identified using tRNAscan-SE 1.21 [61]. Ribosomal RNAs were identified by BLASTN search against Refseq genomes database. TMHMM program with default parameters [62] was used for prediction of transmembrane helices. The BLASTCLUST program [63] set up with a length coverage cutoff of 0.8 and a score coverage threshold (bit score divided by alignment length) of 0.5 was used for clustering of *B. massiliensis* proteins to identify expanded families. The SignalP program [64] was used for prediction of signal peptides. The Marcoil program with default parameters [65] was used for prediction of coiled-coil regions. Genome start was set at the putative Origin of replication which was identified using GCskew analysis [66] and is located 152 nt upstream of the start of the *dnaA* gene.

### Phylogenetic tree construction

The set of 50 conserved bacterial ribosomal proteins [19] from 110 representative genomes and the respective proteins from *B. massiliensis* and JCVI TM6SC1 proteins were aligned by MUSCLE [67] and all alignments were concatenated. Alignment columns containing a fraction of gaps greater than 30% and columns with low information content were removed from the alignment as described previously [68], leaving 6,092 positions in the alignment. A tree constructed using the FastTree program [69] with default parameters (JTT evolutionary model, discrete gamma model with 20 rate categories was used to reconstruct a preliminary maximum-likelihood (ML) tree. ProtTest [70] was used to determine the best substitution model. The optimal substitution model (LG + G) was employed to reconstruct ribosomal protein trees using TreeFinder (1,000 replicates) [71]. The Expected-Likelihood Weights (ELW) of 1,000 local rearrangements were used as confidence values of TreeFinder tree branches. The same model was used for phylogentic reconstruction with RAxML program [72]. For reconstruction of other phylogentic trees based on amino acid sequences we used BLASTCLUST to eliminate sequence redundancy, MUSCLE [67] for sequence alignment (which, in some cases, was corrected manually based on the PSI-BLAST [56] output and FastTree program with default parameters [69]. Further details on phylogenomic analysis are available in Additional file 14.

The 16S RNA sequences were aligned using the SILVA ribosomal RNA gene database project [73]. For 16S RNA phylogeny reconstruction, two maximum likelihood methods, namely FastTree [69] and TreeFinder [71] with the GTRGAMMA model, were employed.

## Referees comments

### **Reviewer comment 1:** Dr Igor Zhulin

**Report form:** This paper reports characterization of an intracellular bacterium *Babela massiliensis* and its genome. 16S rRNA phylogenetics places *B. massiliensis* into a TM6 phylum, for which only one incomplete genome was available prior to this study. An unusually high level of horizontally transferred genes is reported in *B. massiliensis* suggesting that its localized,but rich habitat promotes HGT. Overall, this is an interesting story, and a thorough and well-documented study. Main conclusions are supported by results and the paper is technically sound.

***Authors’ response:****We thank reviewer for this comment.*

**Minor points:**

**Line 68.** At least several environmental isolates of Chlamydiae are known, so strictly speaking they are not “strictly intracellular”.

***Author’s response:****We thank the reviewer for this precision, and as suggested, we modified the sentence: “strictly intracellular” was replaced by “intracellular”.*

Figure 4. Having Deferribacteres in company with Epsilonproteobacteria, whereas Deltaproteobacteria cluster with Acidobacteria, does not add much confidence in this tree. Could some HGT-prone ribosomal proteins account for this noise?

***Authors’ response:****Generally, trees of concatenated universal ribosomal proteins are not known to be substantially affected by HGT. We surmise that these well supported affinities are likely to reflect previously unnoticed evolutionary relationships that are becoming apparent thanks to the increased representation of different bacterial phyla in the genome sequence database. As we point out in the article: “Phylogenetic analysis described here suggests that this phylum might belong to a superphylum together with Acidobacteria, Deferribacter and Proteobacteria.”*

**Quality of written English:** Acceptable

**Quality of Figures:** Acceptable.

### **Reviewer comment 2:** Dr Jeremy Selengut

This work is an important study of an organism that fills in a large gap in our knowledge of the bacterial tree of life.

*B. massiliensis* appears to inhabit a fascinating and distinct biological niche and employ distinctive strategies different from any other organism. Particularly notable are its unusual mode of cell multiplication, its high proportion of genes potentially obtained from other organisms, and a uniquely high number of protein-protein interaction and cell-signalling domains more typical of eukaryotic proteins and often observed in bacterial and viral parasites of amoebae.

As noted by the authors, it is difficult to clearly ascertain the difference between genes that represent horizontal gene transfer (HGT), and those that are merely misplaced in phylogenetic trees based on the deep phylogenetic divergence of *B. massiliensis* and other genome-sequenced organisms. The analysis presented here is sufficient, however, to provide some reasonable support for their hypothesis that extensive HGT is a characteristic of this organism living in the cosmopolitain environment inside an amoebal cell. The best additional tests of this hypothesis will enabled by future sequencing and comparative analysis of other TM6-phylum organisms.

The conclusion that *B. massiliensis* is an obligate intracellular organism is supported by several independent lines of evidence including negative observations of independent growth in typical media, an apparently amoeba-specific lifestyle under microscopy, and most significantly, a severely reduced repertoire of basic metabolic genes.

The mode of intra-amoebal transmission remains to be worked out, along with the amount of time and types of external conditions under which *B. massiliensis* can survive and remain viable after amoebal lysis.

The authors detail the unususal cell multiplication mechanism of *B. massiliensis*, and observe a distinct reduction in the normal set of cell division genes present in free-living organisms – a state more frequently observed in strict intracellular organisms with genomes even more reduced than *B. massiliensis*. The distinct division mode appears to be quite different from these other minimal genome organisms. In particular, measurements of ftsZ gene expression, show distinct differences between *B. massiliensis* and another obligate intra-amoebal pathogen, *L. drancourtii*. The authors suggest the possibility that other unidentified genes may be involved in these processes. A more detailed comparative analysis of the known cell division genes among reduced genomes than was performed here might also reveals important features.

**Quality of written English:** Acceptable

***Authors’ response:****We thank reviewer for these interesting comments, and agree that more detailed analysis are needed to elucidate important features regarding the unique development of B. massiliensis.*

### **Reviewer comment 3:** Pr Martijn Huynen

The manuscript provides a description of the genome, the genomic elements found and the ones conspicuously absent from a newly discovered bacterial species that infects a eukaryotic host. This is a very well written, original manuscript, the observations with respect to the genome content are well put into the context of previous work. The experimental observations about the cell division are fascinating, while the ones about gene expression nicely complement the genome analysis. The pictures of the dividing bacteria within the host are fascinating!

***Authors’ response:****We thank the reviewer for these comments*

One editorial remark: on page 16 is written “(the only one encoded in the genome).” Does that "only one" refer to the only secretion system, or the only type II secretion system.

***Authors’ response:****Yes, this is the only detected secretion system of any type – clarified accordingly in the revised manuscript.*

A second one is that I find the conclusion superfluous, everything in there has already been said in the discussion.

***Authors’ response:****Conclusions is a mandatory section in BMC journals. The text was shortened to reduce the overlap with Discussion.*

## Additional files

Additional file 1:
***Multiplication cycle of*** ***B.massiliensis*** **in the amoebas, stained with Gimenez staining (pictures of the top) and labeled with DAPI (pictures of the bottom), from H0 to H22 post infection, observed with optical microscopy (x100).** Solid arrows indicate mature bacterial particles into the amoebal cytoplasm. Dotted arrows indicate the amorphous growing particles, getting larger during the multiplication cycle. Double arrows indicate amoebal nucleus. Additional file 2:
**Measurement of mature particles and amorphous elements of** ***B. massiliensis*** **during the replication cycle.** Sizes of the growing bacterial particles are observed in the amoebal cytoplasm of *A. polyphaga*, and measured in μm, from H0 to H22 pi. Additional file 3:
**Morphological features of different development satges of the bacteria.** At H0 and H24 post infection, solid arrows point to mature particles included in cytoplasmic vacuoles. At H8 solid arrow shows an amorphous structure strating to grow into a cytoplasmic vacuole. At H15, dotted arrows point to the beginning of the amorphous structure’s segmentation, likely leading to the individualization of mature particles. Additional file 4:
**Features regarding quantification of bacterial multiplication and bacterial effect on amoebae.** **A**: bacterial replication of *B. massiliensis* and *L. drancourtii*. Blue diamond: increase of *B. massiliensis* DNA measured by quantitative PCR. Blue square: increase of the infectious *B. massiliensis* bacterial particles, evaluated by end-point dilution method. Red triangle: increase of *L. drancourtii* measured by quantitative PCR. Red square: growth of *L. drancourtii* evaluated by end-point dilution method. **B**: amoebal pathogenicity of *Babela massiliensis*, the evolution of the amount of amoeba is represented for an uninfected amoebal culture (red bars), and for an amoebal culture infected with *B. massiliensis* (blue bars). **C**: comparison of the amoebal pathogenic effect of *B. massiliensis* and *L. drancourtii,* on the amoebal species *A. polyphaga*. Bacterial growth is measured by quantification of DNA using qPCR, and represented in log[C]. Amoebal lysis is measured by counting the amount of living and dead amoeba per ml. **D**: change of the amount of uninfected *A. polypgaga* in a non-nutritive buffer (PAS) during 24 h. Results are shown in amoeba/ml. Additional file 5:
**Taxonomic distribution and number of the best BLAST hits for** ***B. massiliensis*** **and JCVI TM6SC1.** **A**: Distribution of best hits to different taxa for three groups of *B. massiliensis* proteins. The collection of complete genomes from the Refseq database was used for comparison. The hits were compared using PSI-BLAST bit scores. **B**: The number of best hits to different taxa in the Refseq database for *B. massiliensis* and JCVI TM6SC1. For PSI-BLAST E-value cutoff 1e-5 was used for best hits retrieval and all hits to eukaryotes and archaea were validated manually to eliminate false positives. The scores for JCVI TM6SC1 were obtained in a separate BLAST run against available proteins from the JCVI TM6SC1 genome. Accordingly, the hits in the blue histogram should be interpreted as the second best hits after those to JCVI TM6SC1. Additional file 6:
**Phylogenomics of conserved proteins of** ***B. massiliensis.***
Additional file 7:
**Phylogenomics of** ***B. massiliensis*** **and 10 other bacteria with comparable genome sizes.**
Additional file 8:
**Maximum-Likelihood trees for JCVI TM6SC1 and** ***B. massiliensis*** **proteins with apparent different origins reconstructed using TreeFinder program.** **A**: COG0717: Deoxycytidine deaminase **B**: COG1793: ATP-dependent DNA ligase. Additional file 9:
**Complete annotation of** ***B. massiliensis*** **genes.**
Additional file 10:
**Metabolism of** ***B. massiliensis*** **inferred from genome analysis.**
Additional file 11:
**Comparative genomics of** ***B. massiliensis*** **and selected representatives of parasites of amoeba and bacteria with comparable genome sizes.**
Additional file 12:
**RT-PCR assays assessing the particularity of the cell division machinery of** ***B. massiliensis.*** **A**: expression of FtsZ, FtsA and GroEL genes measured by qPCR, after RT-PCR, for *B. massiliensis*, during 32 hours, every 4 hours. Results are expressed in log cDNA. **B**: expression of FtsZ gene measured by qPCR, after RT-PCR, for *L. drancourtii* and *B. massiliensis*, during 32 hours, every 4 hours. The expression of the gene is compared to the bacterial growth of both microorganisms, measured by end-point dilution method and expressed in log [C]. Additional file 13:
**Identification of 9 closely related and specific to** ***B. massiliensis*** **proteins containing an homolog of the eukaryotic desumoylating Ulp1 peptidase of the C48 family.** **A**. The multiple sequence alignment of selected representatives of Ulp1 peptidase of the C48 family and homologs identified in *B. massiliensis*. The alignments were constructed using the MUSCLE program for Ulp1 peptidases of eukaryotes and *B. massiliensis* homologs separately and then were superimposed on the basis of conserved regions identified by HHpred. The regions identified by HHpred are shown between the alignments and in Figure S10B. Secondary structure for *B. massiliensis* sequences was predicted using the Jpred program. The secondary structure for Ulp1 peptidase from *Saccharomyces cerevisiae,* PDB: 1EUV; YPL020C, is shown underneath the respective sequence. Secondary structure prediction is shown as follows: 'H' indicates α-helix, 'E' indicates extended conformation (β-strand). Conserved catalytic residues are highlighted in red. **B**. HHpred output: pairwise alignments for the *B. massiliensis* gene 300 and selected sequence profiles related to Ulp1 peptidases of C48 family. Additional file 14:
**Additional material and methods.** Additional methods regarding isolation and morphological characterization of *B. massiliensis*; pathogenicity for amoeba; preliminary molecular identification; real-time PCR assays; reverse transcription PCR assays; nucleotide sequences of oligonucleotidic primers and probes used in this study; and phylogenomics of universal bacterial proteins.

## Abbreviations

CDS: Coding DNA Sequence
COGs: Clusters of Orthologous Genes
H0 p.i: Hour zero post-infection
HGT: Horizontal gene transfer
NCLDV: NucleoCytoplasmic Large DNA Viruses
PAS: Page’s Amoebal Salin
PYG: Proteose peptone – Yeast extract – Glucose
