# Supplementary figures and images for: Babela massiliensis, a representative of a widespread bacterial phylum with unusual adaptations to parasitism in amoebae
Source: Biol Direct. 2015 Mar 31;10:13. doi: 10.1186/s13062-015-0043-z (PMC4378268; doi:10.1186/s13062-015-0043-z)

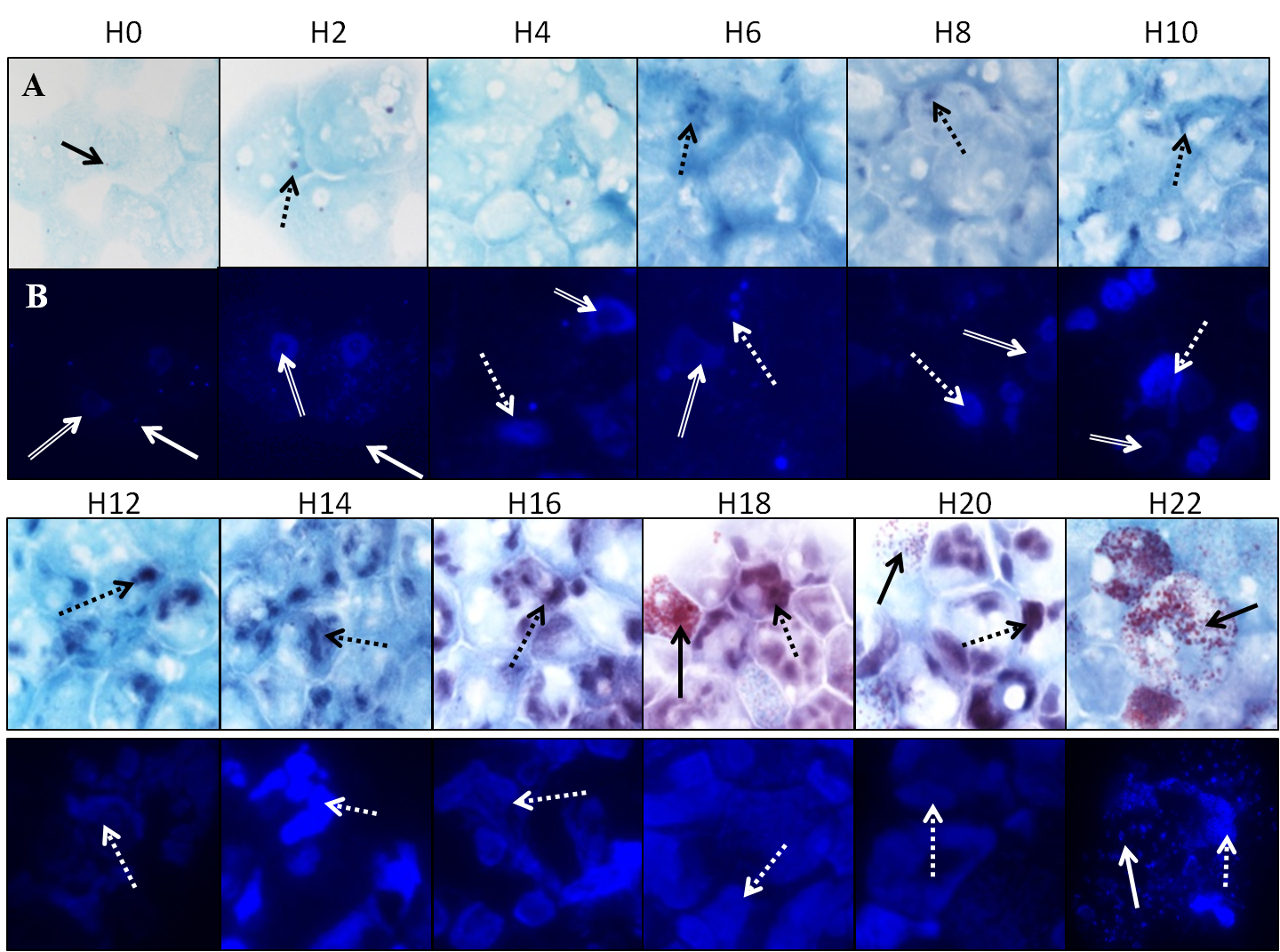

Supplement: Additional file 1: — Multiplication cycle of B.massiliensis in the amoebas, stained with Gimenez staining (pictures of the top) and labeled with DAPI (pictures of the bottom), from H0 to H22 post infection, observed with optical microscopy (x100). Solid arrows indicate mature bacterial particles into the amoebal cytoplasm. Dotted arrows indicate the amorphous growing particles, getting larger during the multiplication cycle. Double arrows indicate amoebal nucleus. [file 13062_2015_43_MOESM1_ESM.png]

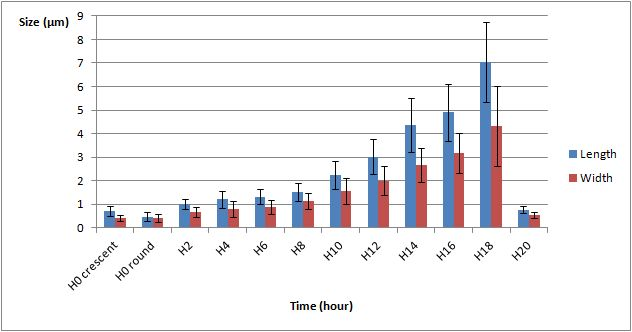

Supplement: Additional file 2: — Measurement of mature particles and amorphous elements of B. massiliensis during the replication cycle. Sizes of the growing bacterial particles are observed in the amoebal cytoplasm of A. polyphaga, and measured in μm, from H0 to H22 pi. [file 13062_2015_43_MOESM2_ESM.png]

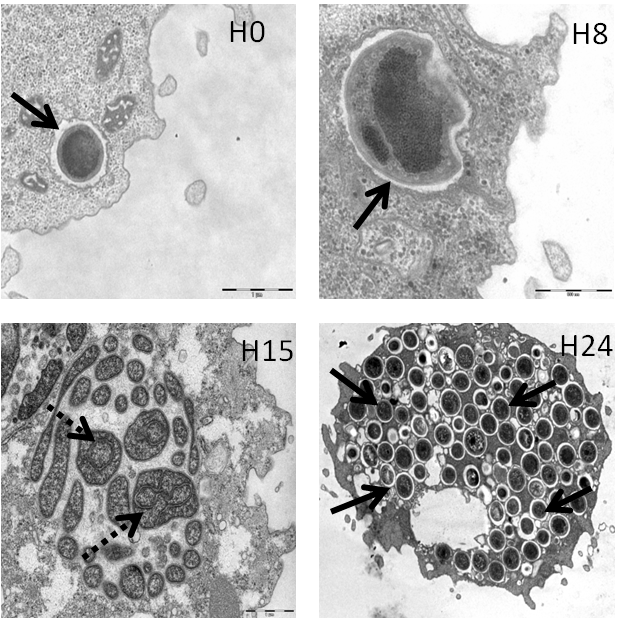

Supplement: Additional file 3: — Morphological features of different development satges of the bacteria. At H0 and H24 post infection, solid arrows point to mature particles included in cytoplasmic vacuoles. At H8 solid arrow shows an amorphous structure strating to grow into a cytoplasmic vacuole. At H15, dotted arrows point to the beginning of the amorphous structure’s segmentation, likely leading to the individualization of mature particles. [file 13062_2015_43_MOESM3_ESM.png]

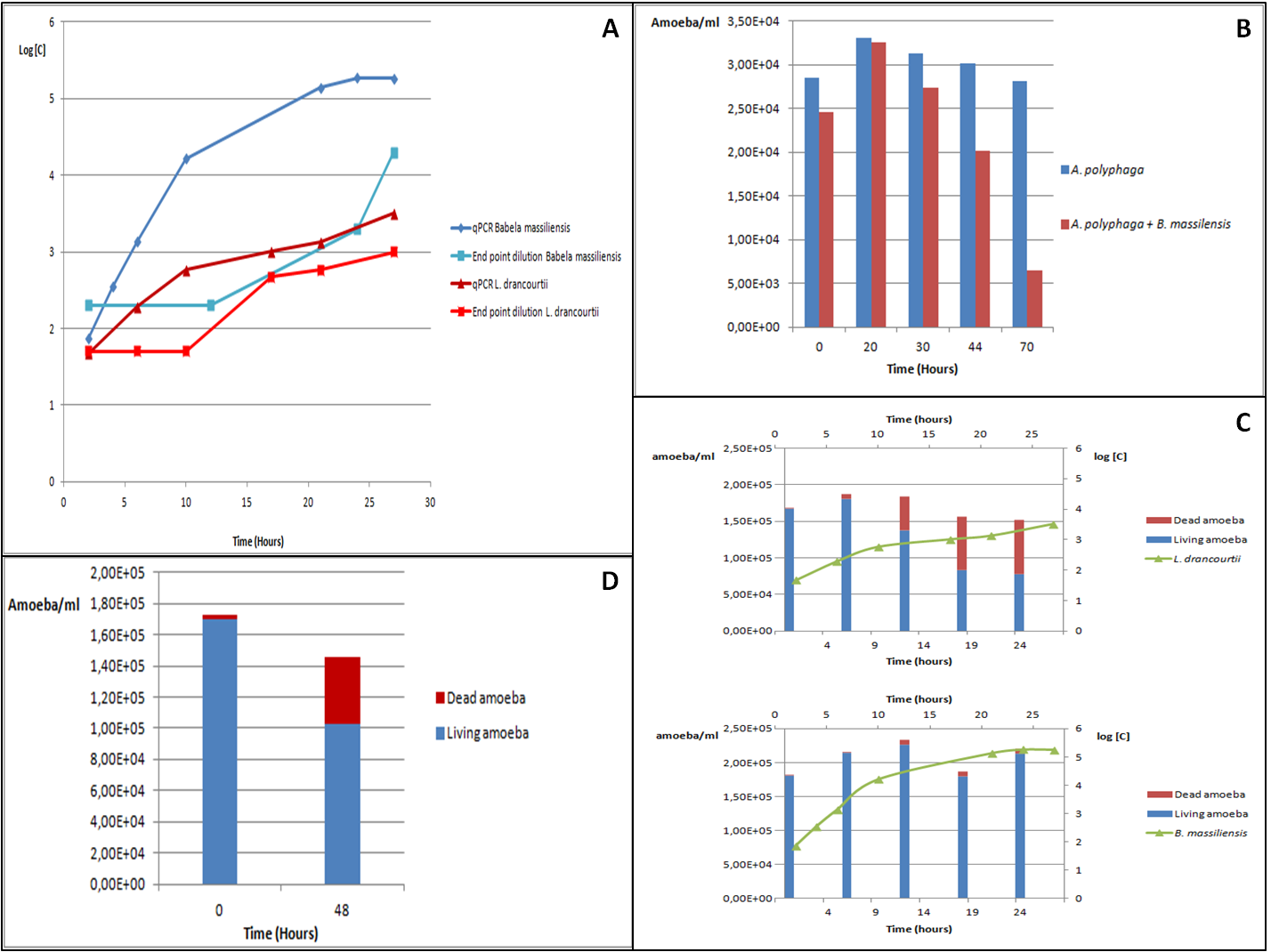

Supplement: Additional file 4: — Features regarding quantification of bacterial multiplication and bacterial effect on amoebae. A: bacterial replication of B. massiliensis and L. drancourtii. Blue diamond: increase of B. massiliensis DNA measured by quantitative PCR. Blue square: increase of the infectious B. massiliensis bacterial particles, evaluated by end-point dilution method. Red triangle: increase of L. drancourtii measured by quantitative PCR. Red square: growth of L. drancourtii evaluated by end-point dilution method. B: amoebal pathogenicity of Babela massiliensis, the evolution of the amount of amoeba is represented for an uninfected amoebal culture (red bars), and for an amoebal culture infected with B. massiliensis (blue bars). C: comparison of the amoebal pathogenic effect of B. massiliensis and L. drancourtii, on the amoebal species A. polyphaga. Bacterial growth is measured by quantification of DNA using qPCR, and represented in log[C]. Amoebal lysis is measured by counting the amount of living and dead amoeba per ml. D: change of the amount of uninfected A. polypgaga in a non-nutritive buffer (PAS) during 24 h. Results are shown in amoeba/ml. [file 13062_2015_43_MOESM4_ESM.png]

A

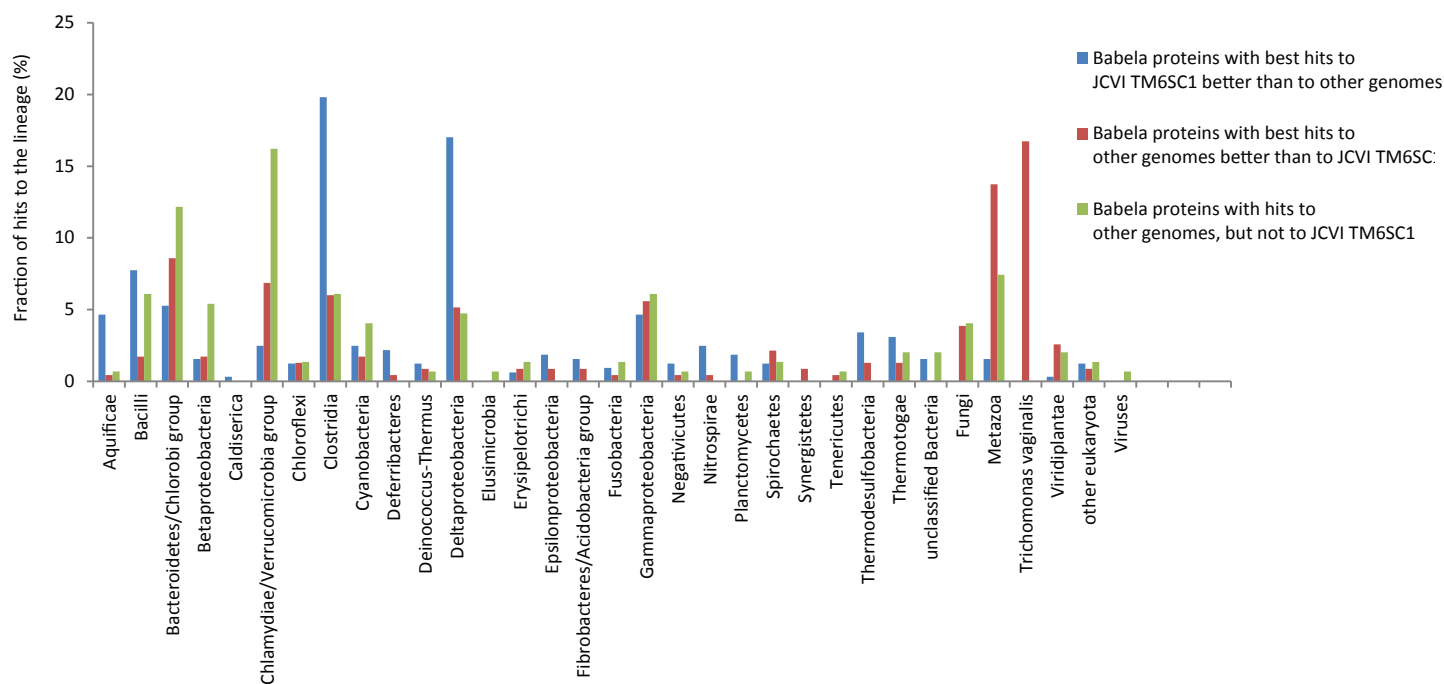

B

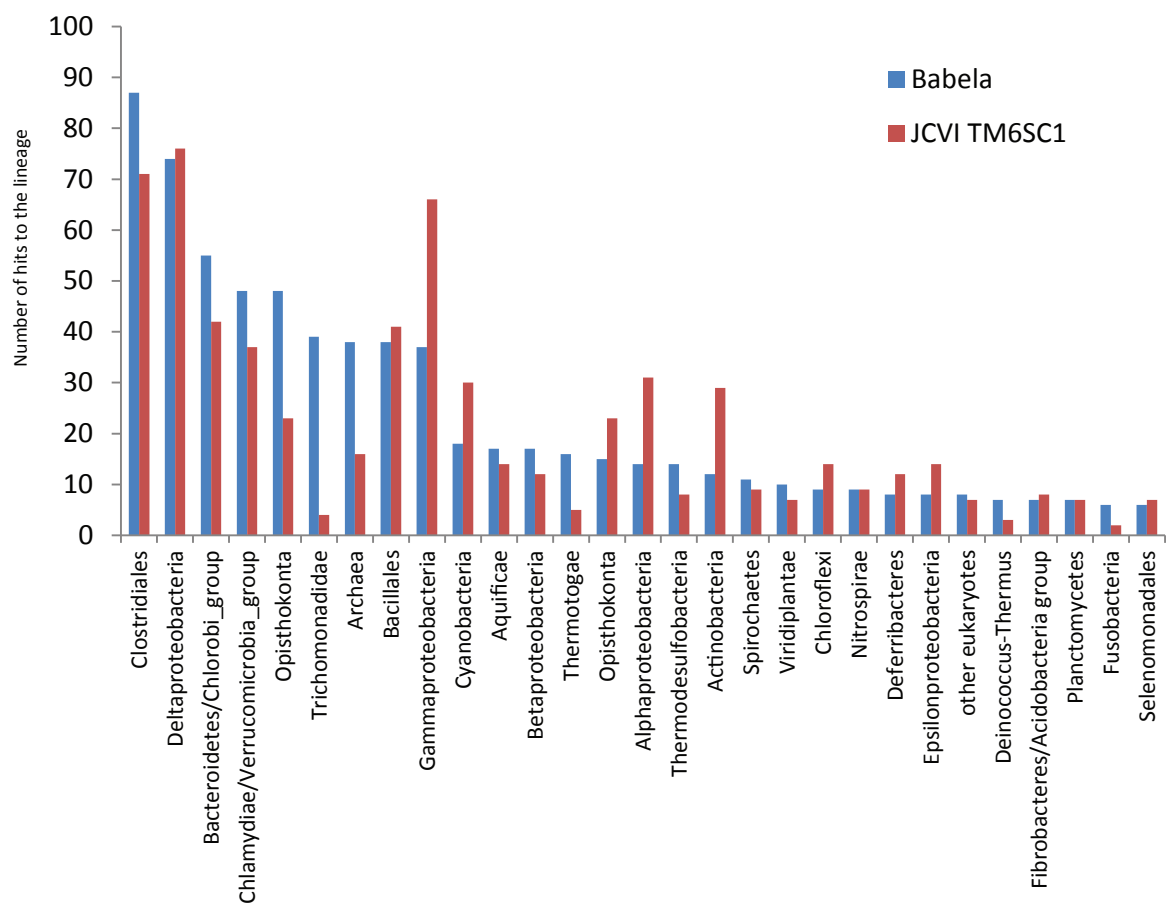

Supplement: Additional file 5: — Taxonomic distribution and number of the best BLAST hits for B. massiliensis and JCVI TM6SC1. A: Distribution of best hits to different taxa for three groups of B. massiliensis proteins. The collection of complete genomes from the Refseq database was used for comparison. The hits were compared using PSI-BLAST bit scores. B: The number of best hits to different taxa in the Refseq database for B. massiliensis and JCVI TM6SC1. For PSI-BLAST E-value cutoff 1e-5 was used for best hits retrieval and all hits to eukaryotes and archaea were validated manually to eliminate false positives. The scores for JCVI TM6SC1 were obtained in a separate BLAST run against available proteins from the JCVI TM6SC1 genome. Accordingly, the hits in the blue histogram should be interpreted as the second best hits after those to JCVI TM6SC1. [file 13062_2015_43_MOESM5_ESM.pdf]

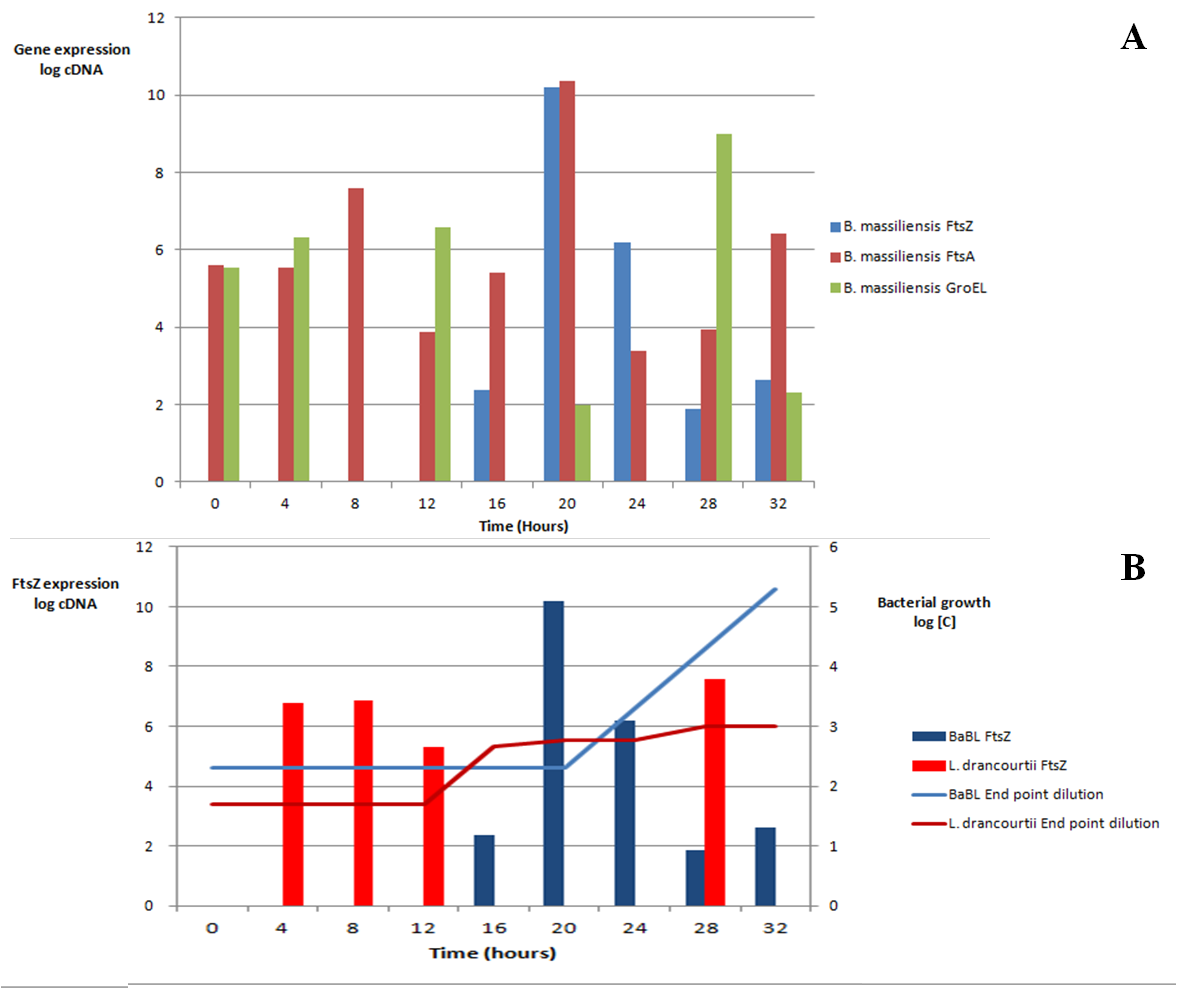

Supplement: Additional file 12: — RT-PCR assays assessing the particularity of the cell division machinery of B. massiliensis. A: expression of FtsZ, FtsA and GroEL genes measured by qPCR, after RT-PCR, for B. massiliensis, during 32 hours, every 4 hours. Results are expressed in log cDNA. B: expression of FtsZ gene measured by qPCR, after RT-PCR, for L. drancourtii and B. massiliensis, during 32 hours, every 4 hours. The expression of the gene is compared to the bacterial growth of both microorganisms, measured by end-point dilution method and expressed in log [C]. [file 13062_2015_43_MOESM12_ESM.png]
