# Supplementary material for: Babela massiliensis, a representative of a widespread bacterial phylum with unusual adaptations to parasitism in amoebae
Source: Biol Direct. 2015 Mar 31;10:13. doi: 10.1186/s13062-015-0043-z (PMC4378268; doi:10.1186/s13062-015-0043-z)
Supplement: Additional file 8: — Maximum-Likelihood trees for JCVI TM6SC1 and B. massiliensis proteins with apparent different origins reconstructed using TreeFinder program. A: COG0717: Deoxycytidine deaminase B: COG1793: ATP-dependent DNA ligase. [file 13062_2015_43_MOESM8_ESM.pdf]

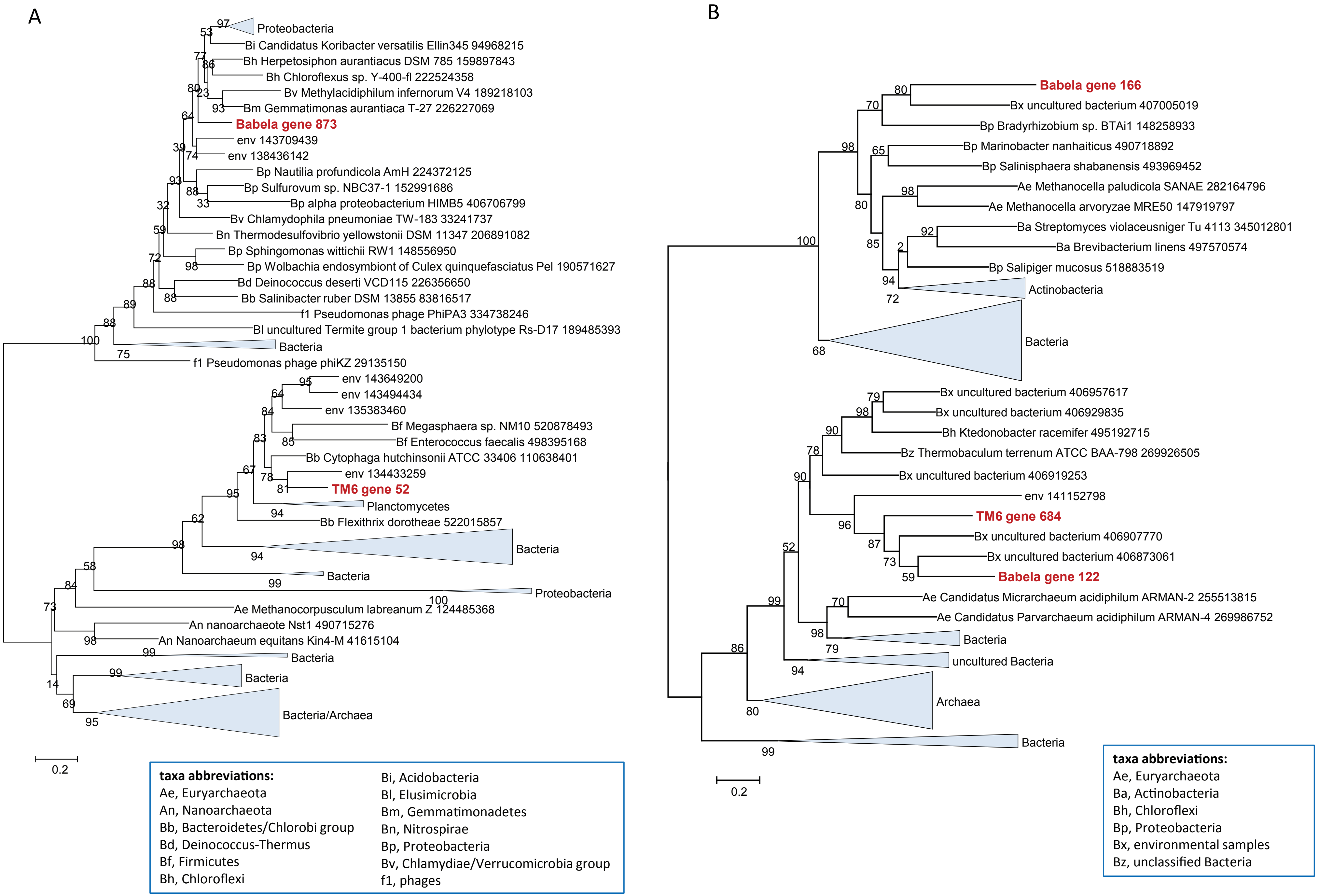
A
Proteobacteria
Bi Candidatus Koribacter versatilis Ellin345 94968215
Bh Herpetosiphon aurantiacus DSM 785 159897843
Bh Chloroflexus sp. Y-400-fl 222524358
Bv Methylacidiphilum infernorum V4 189218103
Bm Gemmatimonas aurantiaca T-27 226227069
Babela gene 873
env 143709439
env 138436142
Bp Nautilia profundicola AmH 224372125
Bp Sulfurovum sp. NBC37-1 152991686
Bp alpha proteobacterium HIMB5 406706799
Bv Chlamydophila pneumoniae TW-183 33241737
Bn Thermodesulfovibrio yellowstonii DSM 11347 206891082
Bp Sphingomonas wittichii RW1 148556950
Bp Wolbachia endosymbiont of Culex quinquefasciatus Pel 190571627
Bd Deinococcus deserti VCD115 226356650
Bb Salinibacter ruber DSM 13855 83816517
f1 Pseudomonas phage PhiPA3 334738246
Bl uncultured Termite group 1 bacterium phylotype Rs-D17 189485393
Bacteria
f1 Pseudomonas phage phiKZ 29135150
env 143649200
env 143494434
env 135383460
Bf Megasphaera sp. NM10 520878493
Bf Enterococcus faecalis 498395168
Bb Cytophaga hutchinsonii ATCC 33406 110638401
env 134433259
TM6 gene 52
Planctomycetes
Bb Flexithrix dorotheae 522015857
Bacteria
Bacteria
Proteobacteria
Ae Methanocorpusculum labreanum Z 124485368
An nanoarchaeote Nst1 490715276
An Nanoarchaeum equitans Kin4-M 41615104
Bacteria
Bacteria
Bacteria/Archaea
0.2
taxa abbreviations:
Ae, Euryarchaeota
An, Nanoarchaeota
Bb, Bacteroidetes/Chlorobi group
Bd, Deinococcus-Thermus
Bf, Firmicutes
Bh, Chloroflexi
Bi, Acidobacteria
Bl, Elusimicrobia
Bm, Gemmatimonadetes
Bn, Nitrospirae
Bp, Proteobacteria
Bv, Chlamydiae/Verrucomicrobia group
f1, phages
B
Babela gene 166
Bx uncultured bacterium 407005019
Bp Bradyrhizobium sp. BTAi1 148258933
Bp Marinobacter nanhaiticus 490718892
Bp Salinisphaera shabanensis 493969452
Ae Methanocella paludicola SANAE 282164796
Ae Methanocella arvoryzae MRE50 147919797
Ba Streptomyces violaceusniger Tu 4113 345012801
Ba Brevibacterium linens 497570574
Bp Salipiger mucosus 518883519
Actinobacteria
Bacteria
Bx uncultured bacterium 406957617
Bx uncultured bacterium 406929835
Bh Ktedonobacter racemifer 495192715
Bz Thermobaculum terrenum ATCC BAA-798 269926505
Bx uncultured bacterium 406919253
env 141152798
TM6 gene 684
Bx uncultured bacterium 406907770
Bx uncultured bacterium 406873061
Babela gene 122
Ae Candidatus Micrarchaeum acidiphilum ARMAN-2 255513815
Ae Candidatus Parvarchaeum acidiphilum ARMAN-4 269986752
Bacteria
uncultured Bacteria
Archaea
Bacteria
0.2
taxa abbreviations:
Ae, Euryarchaeota
Ba, Actinobacteria
Bh, Chloroflexi
Bp, Proteobacteria
Bx, environmental samples
Bz, unclassified Bacteria
