# Supplementary material for: Babela massiliensis, a representative of a widespread bacterial phylum with unusual adaptations to parasitism in amoebae
Source: Biol Direct. 2015 Mar 31;10:13. doi: 10.1186/s13062-015-0043-z (PMC4378268; doi:10.1186/s13062-015-0043-z)
Supplement: Additional file 13: — Identification of 9 closely related and specific to B. massiliensis proteins containing an homolog of the eukaryotic desumoylating Ulp1 peptidase of the C48 family. A. The multiple sequence alignment of selected representatives of Ulp1 peptidase of the C48 family and homologs identified in B. massiliensis. The alignments were constructed using the MUSCLE program for Ulp1 peptidases of eukaryotes and B. massiliensis homologs separately and then were superimposed on the basis of conserved regions identified by HHpred. The regions identified by HHpred are shown between the alignments and in Figure S10B. Secondary structure for B. massiliensis sequences was predicted using the Jpred program. The secondary structure for Ulp1 peptidase from Saccharomyces cerevisiae, PDB: 1EUV; YPL020C, is shown underneath the respective sequence. Secondary structure prediction is shown as follows: 'H' indicates α-helix, 'E' indicates extended conformation (β-strand). Conserved catalytic residues are highlighted in red. B. HHpred output: pairwise alignments for the B. massiliensis gene 300 and selected sequence profiles related to Ulp1 peptidases of C48 family. [file 13062_2015_43_MOESM13_ESM.pdf]

A

B

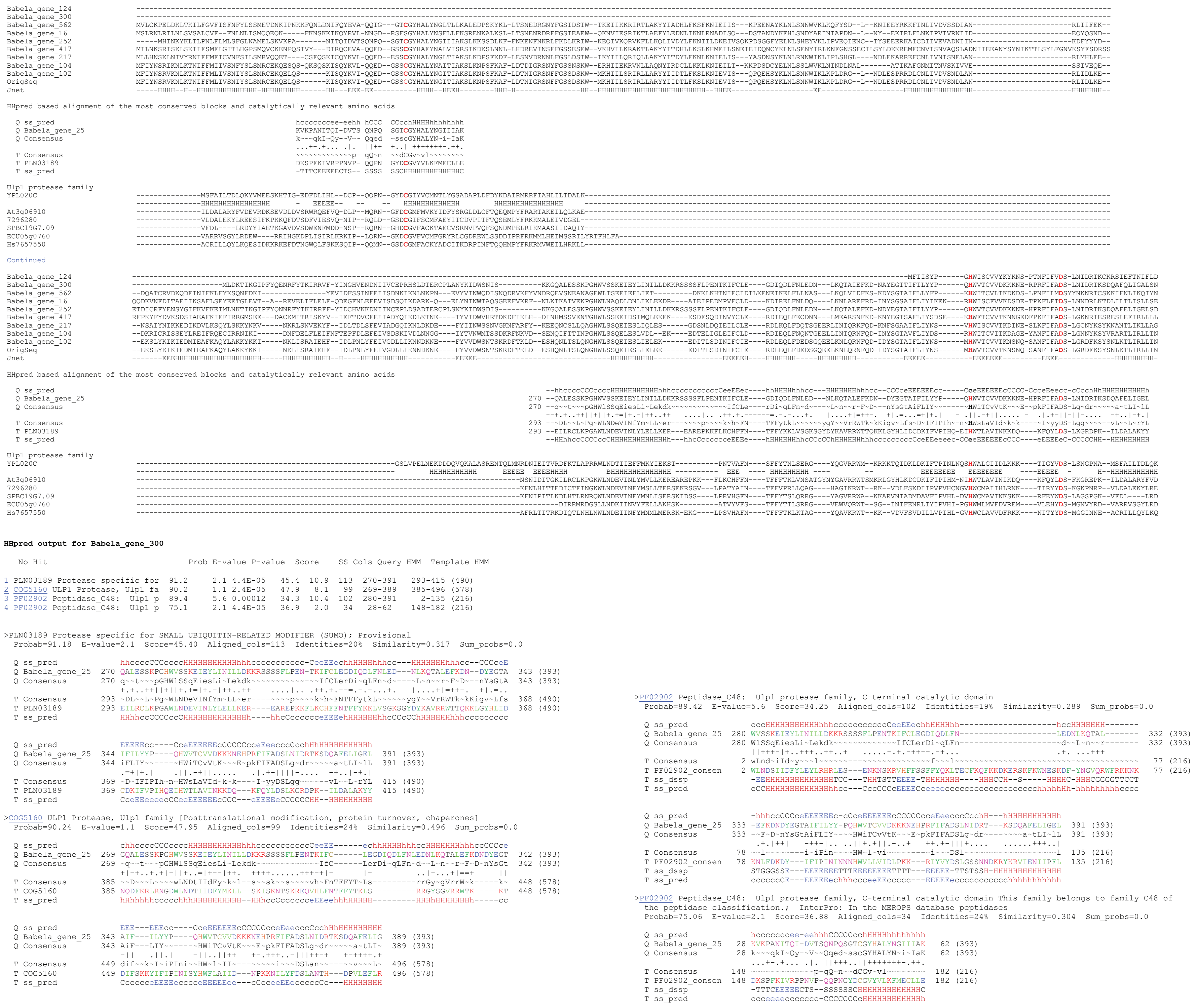

Babela_gene_124
Babela_gene_300
Babela_gene_562
Babela_gene_16
Babela_gene_252
Babela_gene_417
Babela_gene_217
Babela_gene_104
Babela_gene_102
OrigSeq
Jnet
HHpred based alignment of the most conserved blocks and catalytically relevant amino acids
Q ss_pred
Q Babela_gene_25
Q Consensus
T Consensus
T PLN03189
T ss_pred
Ulp1 protease family
YPL020C
At3g06910
7296280
SPBC19G7.09
ECU05g0760
Hs7657550
Continued
HHpred output for Babela_gene_300
No Hit Prob E-value P-value Score SS Cols Query HMM Template HMM
1 PLN03189 Protease specific for 91.2 2.1 4.4E-05 45.4 10.9 113 270-391 293-415 (490)
2 COG5160 ULP1 Protease, Ulp1 fa 90.2 1.1 2.4E-05 47.9 8.1 99 269-389 385-496 (578)
3 PF02902 Peptidase_C48: Ulp1 p 89.4 5.6 0.00012 34.3 10.4 102 280-391 2-135 (216)
4 PF02902 Peptidase_C48: Ulp1 p 75.1 2.1 4.4E-05 36.9 2.0 34 28-62 148-182 (216)
>PLN03189 Protease specific for SMALL UBIQUITIN-RELATED MODIFIER (SUMO); Provisional
Probab=91.18 E-value=2.1 Score=45.40 Aligned_cols=113 Identities=20% Similarity=0.317 Sum_probs=0.0
>COG5160 ULP1 Protease, Ulp1 family [Posttranslational modification, protein turnover, chaperones]
Probab=90.24 E-value=1.1 Score=47.95 Aligned_cols=99 Identities=24% Similarity=0.496 Sum_probs=0.0
>PF02902 Peptidase_C48: Ulp1 protease family, C-terminal catalytic domain
Probab=89.42 E-value=5.6 Score=34.25 Aligned_cols=102 Identities=19% Similarity=0.289 Sum_probs=0.0
>PF02902 Peptidase_C48: Ulp1 protease family, C-terminal catalytic domain This family belongs to family C48 of the peptidase classification.; InterPro: In the MEROPS database peptidases
Probab=75.06 E-value=2.1 Score=36.88 Aligned_cols=34 Identities=24% Similarity=0.304 Sum_probs=0.0
