# Supplementary material for: Babela massiliensis, a representative of a widespread bacterial phylum with unusual adaptations to parasitism in amoebae
Source: Biol Direct. 2015 Mar 31;10:13. doi: 10.1186/s13062-015-0043-z (PMC4378268; doi:10.1186/s13062-015-0043-z)
Supplement: Additional file 14: — Additional material and methods. Additional methods regarding isolation and morphological characterization of B. massiliensis; pathogenicity for amoeba; preliminary molecular identification; real-time PCR assays; reverse transcription PCR assays; nucleotide sequences of oligonucleotidic primers and probes used in this study; and phylogenomics of universal bacterial proteins. [file 13062_2015_43_MOESM14_ESM.docx]

**Additional Methods:**

***Isolation and morphological characterization of Babela massiliensis*.**

Then, 100 µl of this suspension were inoculated onto an amoebal microplate suspension, with 10µl of vancomycin at 10µg/ml (Vancomycin chlorhydrate, Merck) under previously described conditions [1]. Co-cultures were screened every day for cytopathic effect by examination under inverted microscope. After detection of cytopathic effect and confirmation with staining, of the presence of a cocci-like microorganism growing in amoeba, end point dilution method was performed in a 48 microwell plate in order to obtain clonal isolate. The bacterium was then produced as usually by inoculation of supernatants onto fresh amoebal layers in PYG medium. After 48 hours culture, slides were prepared by cytocentrifugation of 100 µl of the co-culture and stained with Gram (Biomerieux), Gimenez [2], methyl alcohol blue, and Hemacolor (Merck) staining. The amoebal culture was centrifugated at 2000 rpm for 10 minutes to pellet infected amoebas. The pellet and the supernatant were separately fixed in a 2,5% glutaraldehyde solution and stained for electron microscopy. The supernatant was also prepared for scanning electron microscopy. Several assays were performed to check the growth capacity of the bacteria without amoeba: nutritive PYG medium, TS broth, Columbia Sheep Blood Agar (Biomerieux), BCYE agar (Oxoid), Chocolate agar (Biomerieux). Different metabolic conditions were tested: four different temperatures ranging from 4°C to 37°C, aerobic, anaerobic, and microaerophilic conditions.

***Pathogenicity for amoeba.*** First, amoebal pathogenicity was evaluated by comparing the amount of amoeba infected with *B. massiliensis*, and the amount of amoeba in a negative control with non infected *A. polyphaga*. Preparation of inoculation was performed as described above, with amoeba rinsed in PAS buffer. Quantification of amoebae was performed in counting slides (HYCOR Glasstic slides) at H0, H20, H30, H44 and H70, till complete lysis of amoeba. Second, we compared the amoebal pathogenic effect of both *B. massiliensis* and *Legionella drancourtii*, by counting the amount of *A. castellanii* remaining alive and dead in the culture, using counting slides (HYCOR Glasstic slides), at H0, H6, H12, H18 and H24. The presence of dead amoeba was objectived with a 0.4% Trypan Blue Solution (Sigma), used in half dilution with the amoebal co-culture. To evaluate the natural decrease of *A. castellanii*  in PAS buffer, a non infected amoebal culture was used as negative control, amoeba were counted at H0 and H24, using counting slides (HYCOR Glasstic slides), also with half volume of 0.4% Trypan Blue Solution (Sigma).

***Preliminary molecular identification.*** For preliminary identification, amplification and sequencing of the complete 16S rRNA gene was performed as described previously [3]. This sequence was compared with sequences available in the GeneBank data base using BLAST program available on the NCBI Web site (<http://www.ncbi.nlm.nih.gov/>), then deposited under GQ495224 Genbank accession number.

***Real-time PCR assays.*** Another intra-amoebal bacterial pathogen, *Legionella drancourtii*, [4], was used as a control, to compare *B. massiliensis* growth in amoeba with a bacteria using a normal binary fission multiplication mode*.* *L. drancourtii* grows preferentially in the amoeba species *A. castellanii*, but not well in *A. polyphaga.* As *B. massiliensis* growth the same way into both amoebal species, assays regarding the comparison between *L. drancourtii* and *B. massiliensis* were processed using the amoebal species *A. castellanii.* The specific *B. massiliensis* DNA increase was evaluated by quantitative PCR, performed with specific primers designed on the 16S rRNA gene with 16SBL564F as forward primer and 16SBL735R as reverse primer, and the TaqMan probe 16SBL631. The increase of the mature bacterial particles was evaluated by end-point dilution method, at each time of the cycle; cultures were sampled at H2, H4, H6, H10, H20, H24 and H27. For *L. drancourtii,* cultures and developmental cycle of the bacteria in amoeba were performed as described above for *B. massiliensis* with the difference that mature bacterial particles were evaluated by counting CFU on BCYE agar plates (Oxoid). The specific *L. drancourtii* DNA increase was evaluated by quantitative PCR performed with specific primers designed in the *mip* gene with mipdrancR for the reverse primer, mipdrancF for the forward primer (see Table below), and SYBR Green (Roche) for detection of the bacterial growth. For *L. drancourtii*, cultures were sampled at H2, H6, H10, H17, H21 and H27*.*

***Reverse Transcription PCR assay.*** Bacterial cultures were performed in PAS buffer, as previously described for real-time PCR essays. Co-cultures of *L. drancourtii* and *B. massiliensis* were performed in *A. castellanii*, and at H0, H6, H12, H18, H24, H28 and H32 post-infection, 20ml of each culture was sampled. Each sample was centrifugated at 5500 rpm for 30 minutes, in order to pellet both infected amoeba and extracellular bacteria. RNA extraction was performed with QiaGen RNA extraction kit, according to manufacturer’s instructions. Total RNA was reverse transcribed using the MMLV-RT kit according to the manufacturer's protocol (Invitrogen), and primers and probes specific to FtsA, FtsZ and GroEL genes were designed using Primer3 free web software (see table below), for both *B. massiliensis* and *L. drancourtii*. For each microorganism and each gene, Real Time PCR was performed using Light Cycler (Roche).

All primers and probes used for Preliminary molecular identification, Real-time PCR assays, and Reverse Transcription PCR assay are provided in the Table below:.

***Nucleotide sequences of oligonucleotide primers and probes.***

| Microorganisme | Gene | | Primer name | Primer sequence |
| --- | --- | --- | --- | --- |
|  |  | |  |  |
| *B. massiliensis* | 16S rRNA | 16SBL564F  16SBL735R  16SBL631 | | Forward primer: 5’ ATCAGCAGTAGATACTGGTA 3'  Reverse primer: 5’ CAGGGTATCTAATCCTGTTT 3'  TaqMan probe: 6 FAM-ATGCGTAGATCTCAAGAGGAAC-TAMRA |
|  | *GroEL* | BGroEL1F  BGroEL1R | | Forward primer : 5’CTG-GTG-GTG-GTG-TTG-CAT-TA 3’  Reverse primer : 5’TCC-AAT-TCC-AGG-TTC-CTC-TG 3’ |
|  | *FtsA* | BFtsA2F  BFtsA2R | | Forward primer : 5’ TCA-AGT-GGA-ATT-AGC-GCA-AG 3’  Reverse primer : 5’ CCG-CCT-GTT-AAA-ACA-AGA-CC 3’ |
|  | *FtsZ* | BFtsZ1F  BFtsZ1R | | Forward primer : 5’ AAC-ACC-GAT-GCT-CAA-GCT-CT 3’  Reverse primer : 5’ GCC-TCC-AAG-ACC-TGC-TGT-TA 3’ |
| *L. drancourtii* | *mip* | MipdrancF  MipdrancR | | Forward primer: 5’ GTCTACTGCAATGGCTGCTG 3’  Reverse primer: 5’ CCATCTTGCATTCCTTTTGC 3’ |
|  | *FtsZ* | DFtsZ2F  DFtsZ12R | | Forward primer : 5’AGT-TGA-GCA-CAT-GGT-TGC-AG 3’  Reverse primer : 5’AAA-GAC-CAT-ATC-CGC-ACC-AG 3’ |
|  | *FtsA* | DFtsA1F  DFtsA1R | | Forward primer : 5’CGT-CAT-CCT-TCT-CGT-GGT-TT 3’  Reverse primer : 5’ATC-AGC-TTG-CGA-CAC-CTC-TT 3’ |
|  | *GroEL* | DGroEL1F  DGroEL1R | | Forward primer : 5’AGA-GTT-TGG-AAG-CGG-CTA-CA 3’  Reverse primer : 5’GCA-ACA-CCG-CCA-GAT-AAT-TT 3’ |
| *Chlamydia* sp. | 16S rRNA | 16SIGF  16SIGR | | Forward primer: 5’CGGCGTGGATGAGGCAT3’  Reverse primer: 5’TCAGTCCCAGTGTTGGC 3’ |
|  | 16S-23S rRNA | 16SF2  23SIGR | | Forward primer: 5’CCGCCCGTCACATCATGG 3’  Reverse primer: 5’ TGGCTCATCATGCAAAAGGCA 3’ |

***Phylogenomics of universal*** ***bacterial proteins***

To assess phylogenetic affiliation of universal bacterial proteins present in *B. massiliensis* genome, the following procedure was developed. A representative set of 506 bacterial genomes (largest genome of each genus plus *E.coli* K12 and *B.subtilis* str. 168 was chosen using NCBI genomes (<http://www.ncbi.nlm.nih.gov/genome/browse/>). A protein (COG) was defined as universal if it was present in at least 95% of the 506 representative bacterial genomes. 219 universal COGs were found. 165 of these COGs were represented in *B. massiliensis* genome. In three cases (namely, excinuclease ABC subunit B, membrane GTPase LepA, and metalloprotease involved in rRNA maturation), *B. massiliensis* proteins were more similar to eukaryotic or archaeal than to bacterial proteins, suggesting non-orthologous gene replacement in *B. massiliensis* genome; these *B. massiliensis* proteins were excluded from the universal bacterial protein analysis.

219 universal COG proteins (with *B. massiliensis* sequences added whenever present) were aligned by MUSCLE; the alignments were refined as described above; ML phylogenetic trees were built using Fasttree. The trees were rooted using a version of mid-point procedure; for each tree, branches related to *B. massiliensis* as ‘sister-to’, ‘rooted-by’, and ‘belongs-to”, were recorded. The results are in Supplementary Table S1 “Phylogenomics of *B. massiliensis* and 10 other bacteria with comparable genome sizes”. To validate the approach, ten other bacteria out of the 506 representatives were chosen and subjected to the same analysis.

**References**

1. La Scola B, Mezi L, Weiller PJ, Raoult D : **Isolation of *Legionella anisa* using an Amoebic Coculture procedure**. *J Clin Microbiol* 2001, **39**: 365-366.
2. Gimenez DF: **Staining *Rickettsiae* in Yolk-sac Cultures**. *Stain Technol* 1964, **39**: 135-140.
3. **La Scola, B., R.J. Birtles, M.N. Maller, and D. Raoult**. 1998. *Massilia timonae* gen. nov., sp.nov., isolated from blood of an immunocompromised patient with cerebellar lesions. J Clin Microbiol **36**(10):2847-2852.
4. **La Scola, B., R.J. Birtles, G. Greub, T.J. Harrisson, R.M. Ratcliff, and D. Raoult.** 2004. *Legionella drancourtii* sp. nov., a strictly intracellular amoebal pathogen. Int J Syst Evol Microbiol **54**(3):699-703.
